# Complementary authentication of Chinese herbal products to treat endometriosis using DNA metabarcoding and HPTLC shows a high level of variability

**DOI:** 10.3389/fphar.2023.1305410

**Published:** 2023-12-05

**Authors:** Felicitas Mück, Francesca Scotti, Quentin Mauvisseau, Ancuţa Cristina Raclariu-Manolică, Audun Schrøder-Nielsen, Helle Wangensteen, Hugo J. de Boer

**Affiliations:** ^1^ Section for Pharmaceutical Chemistry, Department of Pharmacy, University of Oslo, Oslo, Norway; ^2^ Department of Pharmaceutical and Biological Chemistry, UCL School of Pharmacy, University College London, London, United Kingdom; ^3^ Natural History Museum, University of Oslo, Oslo, Norway; ^4^ Stejarul Research Centre for Biological Sciences, National Institute of Research and Development for Biological Sciences, Piatra Neamț, Romania

**Keywords:** chemical fingerprinting, DNA metabarcoding, endometriosis, pharmacovigilance, Traditional Chinese Medicine, women’s healthcare

## Abstract

Traditional Chinese Medicine (TCM) is popular for the treatment of endometriosis, a complex gynecological disease that affects 10% of women globally. The growing market for TCMs has yielded a significant incentive for product adulteration, and although emerging technologies show promise to improve their quality control, many challenges remain. We tested the authenticity of two traditional Chinese herbal formulae used in women’s healthcare for the treatment of endometriosis, known as *Gui Zhi Fu Ling Wan* (FL) and *Ge Xia Zhu Yu Tang* (GX). Dual-locus DNA metabarcoding analysis coupled with high-performance thin-layer chromatography (HPTLC) were used to authenticate 19 FL and six GX commercial herbal products, as well as three *ad hoc* prepared artificial mixtures. HPTLC was able to detect most of the expected ingredients via comparative component analysis. DNA metabarcoding was able to detect an unexpected species diversity in the products, including 38 unexpected taxa. Chromatography has a resolution for all species indirectly through the identification of marker compounds for the different species ingredients. Metabarcoding on the other hand yields an overview of species diversity in each sample, but interpretation of the results can be challenging. Detected species might not be present in quantities that matter, and without validated quantification, some detected species can be hard to interpret. Comparative analysis of the two analytical approaches also reveals that DNA for species might be absent or too fragmented to amplify as the relevant chemical marker compounds can be detected but no amplicons are assigned to the same species. Our study emphasizes that integrating DNA metabarcoding with phytochemical analysis brings valuable data for the comprehensive authentication of Traditional Chinese Medicines ensuring their quality and safe use.

## Introduction

Traditional Chinese Medicine (TCM) is a holistic medical system with a long history of use in healthcare, disease prevention, diagnosis, and treatment, and one of the most popular health resources throughout the world (World Health Organization, 2022). The use of TCMs has gained global prominence and is based on maintaining a balance of vital life force, called *Qi*, which is supposed to surge along meridians in the body and maintain a person’s health. Key components of TCM include *inter alia*, acupuncture, herbal medicine, dietary therapy, movement, and concentration exercises (e.g., *Qi Gong*, *Tai Chi*). TCM has been practiced for over 3000 years and is continuously refined through treatment observations and clinical studies (Lu et al., 2012; Gu and Pei, 2017). As part of TCM practice, Chinese Herbal Medicine (CHM) is regarded as effective for various diseases and believed to cause minimal adverse reactions (Tsou et al., 2022). A recent large-scale randomized and placebo-controlled trial confirmed the applicability of specific CHM as a treatment for endometriosis-associated pain and related symptoms (Lin et al., 2022). This opens interesting alternative treatment options, as conventional treatments might not have desirable effectiveness and may have side effects (Ilhan et al., 2019; Meresman et al., 2021; Taylor et al., 2021; Barnard et al., 2023). ​​Today, TCMs are readily available in shops, in TCM stores, sold as food supplements, and are broadly available through online marketing, and increasingly applied as self-care strategies and self-medication for primary disease patterns in women’s healthcare (Chen et al., 2019; Barnard et al., 2023). The international market and unregulated trade make TCMs vulnerable to fidelity issues in various stages of the supply chain (Xu et al., 2019; Zhu et al., 2022). Concern has been paid to potential health risks and hazards of poor quality and adulterated herbal medicines associated with TCMs, but also to intrinsic toxicities or extrinsic harmful residues detected in a large number of TCMs (Chen et al., 2023; Coghlan et al., 2015; Coghlan et al., 2012; Liu et al., 2020; Liu et al., 2015; Sakurai, 2011; Xu et al., 2019; Zhu et al., 2021). Chinese herbal remedies are usually complex mixtures of multiple ingredients derived primarily from plants, as well as fungi, animals, and minerals, and the result of manifold processing steps (Guo et al., 2015; Liu, 2015; Engelhardt et al., 2018; Wu et al., 2018). This contributes to implication issues for the standardization of herbal products. Furthermore, juristic and marketing differences among countries contribute to poor regulation and subsequent difficulties in quality assurance of marketed herbal products (Low et al., 2017; Raclariu et al., 2018a; Heinrich et al., 2019; Alostad et al., 2020; Thakkar et al., 2020; Ichim and Booker, 2021; Raclariu-Manolică et al., 2023a).

There is an urgent need for the development of rapid and simple inspection procedures and authenticity testing of Chinese herbal materials (Zhu et al., 2022). DNA barcoding systems adopted by various national pharmacopeias, including the Chinese Pharmacopoeia, British Pharmacopoeia, and Japanese Pharmacopoeia (Chen et al., 2017), are applicable for the identification of raw botanical material, but their efficacy is limited in assessing highly processed polyherbal products (Leong et al., 2020). DNA metabarcoding has shown a high resolution to simultaneously confirm both the presence of target species and off-label ingredients that occur in commercial herbal products, such as CHM remedies (Coghlan et al., 2012; Raclariu et al., 2018b; Seethapathy et al., 2019; Anthoons et al., 2021; Zhu et al., 2022; Raclariu-;Manolică et al., 2023a). Multi-locus DNA barcoding has been used in many studies for the authentication of species in TCMs and other traditional medicines (Arulandhu et al., 2017; Arulandhu et al., 2019; Yao et al., 2022; Zhu et al., 2022). Chromatographic fingerprints are known to provide a high resolution for the detection of target compounds of known ingredients and are a well-applied, basic authentication tool for herbal remedies such as TCMs (Liang et al., 2010; Booker et al., 2016; Raclariu et al., 2017; Fitzgerald et al., 2019; Heinrich et al., 2022). Furthermore, fingerprinting techniques can allow the consideration of the complexity of herbal products by evaluating the whole chemical profile and extracting a common pattern to be used as a criterion for elaborating the individual formulation (Noviana et al., 2022).

In this study, we tested the authenticity of two complex TCM formulae used in the treatment of endometriosis, known as *Gui Zhi Fu Ling Wan* (FL) and *Ge Xia Zhu Yu Tang* (GX). FL is one of the most widely known TCM formulae originally composed by Zhang Zhongjing (150–2019 CE), and once published in the oldest clinical book, *Essentials from the Golden Cabinet*, dedicated to internal, external, gynecological and obstetrical diseases during the Eastern Han dynasty (Zhang et al., 2020). Its efficacy has been tested in clinical practice and it is used to treat conditions like endometriosis, uterine fibroids, and pelvic inflammation among others (Fang et al., 2012; Li et al., 2019a; 2019b; 2019c; Gao et al., 2020; Du et al., 2021). The formula includes five Chinese herbal ingredients: Cinnamomi Ramulus, Poria Cocos, Paeoniae Radix Rubra, Moutan Cortex, and Persicae Semen.

The second formula analyzed, GX, originates from the Wang Qing-ren’s dynasty and was first published in 1830, *Correction of Errors among Physicians*. Today, it is also known as the Tangkuei & Corydalis Combination and is specifically used to “drive out stasis below the diaphragm/the Mansion of Blood” (Bensky et al., 2004; Scheid et al., 2009). This formula is more complex and includes 11 to 12 different TCM ingredients, with the 12th being uncommon in products on the European market: Angelica Sinensis Radix, Moutan Cortex, Paeoniae Radix Rubra, Persicae Semen, Linderae Radix, Corydalis Rhizoma, Chuanxiong Rhizoma, Radix et Rhizoma Glycyrrhizae/Glycyrrhiza Radix, Cyperi Rhizoma, Carthami Flos, Aurantii Fructus, and Trogopterori Faeces.

It is worth noting that the official names of TCM ingredients identify the drug through the plant’s name and the part of the plant used, but the ingredient name does not often align with official taxonomy. One of the consequences is that, frequently, one ingredient, e.g., Paeonia Radix (translated as “Peony root”), might be associated with materials coming from more than one single species of *Paeonia*, and is traded as a product with a vernacular name only not matching one-to-one with a scientific plant name. The Chinese Pharmacopoeia reports the latest definitions of which species are accepted under the ingredient name with scientific taxonomy (Chinese Pharmacopoeia Committee, 2020). Yet, there are multiple alternate species, local variants, and possible substitutions that are not coherent with the ingredient monographs of the Chinese Pharmacopoeia, and which are cultivated for TCM purposes. These alternatives are mostly traded locally in China and not marketed internationally (Leon and Yu-Lin, 2017).

The aim of this study was to assess the complementarity of results from integrating dual-locus DNA metabarcoding analysis with high-performance thin-layer chromatography (HPTLC) for the authentication of multi-herbal products, like TCMs.

## Materials and methods

### Sample collection and preparation

Twenty-five samples (*n* = 19 FL and *n* = 6 GX) were collected in 2019 from commercial TCM distributors and online retailers in Europe, as well as from China Town, London (United Kingdom). Samples were sold as mixed powdered TCM herbs (*n* = 3), tablets (*n* = 14), capsules (*n* = 1), pills (*n* = 2), extract and crude powder (*n* = 1), and granules (*n* = 4). Three mixtures (AMs) were prepared *ad hoc*, in the laboratory, using raw materials purchased from a German pharmacy and a Chinese health shop in China Town, London. AMs were prepared from crude herbal materials and received the following identification codes: AM1_FL, AM2_FL, and AM_GX. All ingredients were previously studied for substitutions (Bensky et al., 2004; Leon and Yu-Lin, 2017; Scheid et al., 2015; 2009). Accepted binomial names were retrieved from an automated comparison of 36 databases (AnAge, 2023; AskNature, 2023; Biological Library et al., 2012; Collaborative Collection Managment Solutions, 2023; EOL, 2023; EEA, 2019; FADA database, 2023; GBIF Secretariat, 2023; GISD, 2023; iNaturalists, 2023; Index Fungorum, 2023; ION, 2023; IPNI- The International Plant Names Index, 2023; IRMNG, 2023; ITIS, 2023; IUCN, 2022; NCBI, 2023; Noé, 2011; NLBIF, 2023; NYBG.org, 2023; Open Tree of Life Reference Taxonomy, 2023; Papahanaumokuakea Marine National Monument, 2023; P; Mollel, 2011; Qian et al., 2021; Rapid Biological Inventories, 2023; Sequeira, 2015; Shao and Chung, 2022; Teisher and Stimmel, 2023; The Catalogue of Life, 2023; uBio NameBank, 2023; US Wildflower’s Database of Wildflowers for Illinois, 2023; USDA NRCS PLANTS Database, 2023; VASCAN, 2023; Wikispecies, 2023; Wilson and Reeder, 2005; WORMS- World Register of Marine Species, 2023). The accepted scientific names of the plant species used as ingredients and names of other putative substitute ingredients were validated using the online platform World Flora Online (WFO) (See Supplementary Tables S1A, B). The TCM products were imported into Norway for scientific analyses under Norwegian Medicines Agency license ref. No 18/13493-2. An overview of the TCM products can be found in Supplementary Table S2.

The sample materials were ground and homogenized using an IKA Tube Mill 100 (IKA-Werke GmbH & Co. KG, Staufen, Germany). Artificial mixtures of FL and GX were prepared according to standard ingredient dosing (Scheid et al., 2015). For FL, we mixed 1 g of crude herbal material for each of the five ingredients from two different sample sets in which all single ingredients are derived from a single distributor of CHMs in Europe. This is equivalent to 10% of the standard prescription. Accordingly, we mixed 10% of the crude material of each plant ingredient for GX. For DNA metabarcoding we used three technical replicates for all three mixtures prepared *ad hoc* (AM1_FL: 1-3FL; AM2_FL: 23FL and 24FL and AM_GX: 7-9GX). (See Supplementary Table S3).

### Library preparation and dual locus metabarcoding for TCM authentication

The DNA was extracted using the E.Z.N.A.^®^ SP Plant DNA kit (SKU:D5511, Omega Biotek Inc., Norcross Georgia). The manufacturer’s instructions were followed except for a larger quantity of starting material (up to 30 mg), an elongated lysis step, and larger volumes of buffer in all steps before DNA binding to HiBind column (e.g., 1.6 mL SP1 buffer at 65°C for 1 h) as well as a final elution volume of 100 μL per sample. Dual index fusion primers of the internal transcribed spacers nrITS1 and nrITS2, based on 18S-ITS1F and 58S-ITS1R (Omelchenko et al., 2019), and ITS2F and ITSp4 primers (Timpano et al., 2020), were used to create amplicon libraries. Expected amplicon sizes were 400 bp for nrITS1 and 450 bp for nrITS2. Polymerase chain reactions (PCR) were carried out in 25 μL reactions consisting of 5 μL of template DNA, 1X of AccuStart II PCR ToughMix (AccuStart, Quantabio, Massachusetts, United States), and 0.16 µM of each primer. The PCR cycling protocol consisted of initial denaturation at 94°C for 3 min, followed by 35 cycles of denaturation at 94°C for 10 s, annealing at 52°C for 15 s for ITS1, and at 56°C for 15 s for ITS2, and elongation 72°C for 1 min followed by a final elongation step at 72°C for 2 min. PCRs were conducted using indexed primers as in (Raclariu-Manolică et al., 2023b) following the indexing strategy of (Fadrosh et al., 2014)). All amplicons were visually checked using gel electrophoresis and normalized based on values from the quantity tool in the Image Lab 6.0 software (Bio-Rad Laboratories, Inc., United States). The different genetic markers were kept separate during the normalization and pooling of amplicons. Each pool was cleaned and concentrated using Ampure XP (Beckman Coulter, Inc., United States), and size was selected using a Blue Pippin (Sage Science, Inc., United States) with a size selection window targeting the amplicon size of the respective markers. Then, the pools were visualized on a Fragment Analyzer (Agilent Technologies, Inc.) to verify amplicon length and sent for sequencing on an Illumina MiSeq at the Norwegian Sequencing Centre.

### Bioinformatics analysis

Bioinformatic processes for the metabarcoding analysis were conducted as in Raclariu-Manolică et al. (2023b) using the annotated scripts provided in: https://otagomohio.github.io/workshops/eDNA_Metabarcoding. In brief, forward and reverse raw sequencing files obtained from MiSeq sequencing were merged using PEAR 0.9.3 (Zhang et al., 2014) and subsequently demultiplexed using the ngsfilter command from the OBITools software suite (Boyer et al., 2016). Obigrep command was used to choose fragment sizes of respective barcode regions. Quality filtering was conducted to remove sequences <100 bp and >420 bp for ITS1, <100 bp, and >450 bp for ITS2 primers by using the fastq_filter command from USEARCH algorithm (Edgar, 2010). Afterward, sequences were dereplicated using the fastx_uniques command from the USEARCH algorithm (Edgar, 2010). Sequences with less than 10 occurrences in the dataset were removed. By using the UNOISE algorithm (e.g., unoise3 command from USEARCH) (Edgar, 2016), the dataset was denoised and OTUs (Operational Taxonomic Units) were determined. As a last step, the taxonomic assignment was conducted using the blastn command from the BLAST + application (Camacho et al., 2009). Strict filtering controls were conducted to delete any false positive detections for each sample. For each obtained OTU, we subtracted the highest number of reads that could be found in the corresponding OTU in any of all negative controls (extraction blanks and PCR controls). This standard approach was applied in all PCR replicates of the samples. We used this approach to make sure that potential contaminants or “tag-jumps'' will not result in potential false positives. In each unique sample, only OTUs showing ≥ 5 reads were retained for further analysis. For the taxonomic assignment, only the top-scored species were selected as the target species (search tool: BLAST), and all corresponding species with relative abundance below 0.002 were excluded (Yao et al., 2022). OTUs were checked for species delineation with ASAP: assembling species by automatic partitioning (Puillandre et al., 2021). Hence, OTUs corresponding to the same unique species were pooled together in a unique species identifier, and the read numbers were pooled. Thus, overinflation of the observed species range could be avoided. The relative abundance of each plant species per sample was calculated and a complete overview of the detected species and the corresponding number of genetic sequences/reads is provided in Supplementary File S1. Analysis of the results is presented in figures made using the R packages ggplot and gtools.

### High-performance thin-layer chromatography (HPTLC)

HPTLC marker compounds and other chemicals were obtained from Merck (Darmstadt, Germany) and Sigma-Aldrich (St. Louis, MA, United States), and botanical reference standards were obtained from ChemStrong Scientific Co., Ltd. China. Marker compounds and botanical reference standards were prepared according to Hong Kong Chinese Materia Medica guidelines (HK Chinese Materia Medica, 2021) unless stated differently. Each botanical reference sample was prepared individually. Each herbal ingredient in the formulae was tested using a plant-specific method (see Supplementary Methods S1). All formulas tested were prepared by dissolving 0.50 g in 10 mL of ethanol, followed by sonication for 20 min and filtration using Merck Millex PES syringe filters (0.22 μm). Each TLC plate (silica gel 60 F_254_ Merck KGaA, Darmstadt, Germany) was visualized under white light and UV 254 and 366 nm prior to the commencement of the analytical procedure. Standard solutions and the extracts obtained from the samples and herbal references were spotted in bands of 8.0 mm width, using a CAMAG Linomat 5 instrument (Camag, Muttenz, Switzerland). The bands were applied at a distance of 8.0 mm from the lower edge of the plate and 20 mm from the left edge. Each plate was developed using a tailored method (see Supplementary Methods S1), using CAMAG Automatic Developing Chamber (ADC 2). For derivatization, CAMAG derivatizer was used when the derivatizing reagent complied with CAMAG’s guidelines, otherwise, manual spraying was employed. Following development and derivatization, plates were visualized under white light, UV 254 nm, and 366 nm using CAMAG’s Visualizer (Muttenz, Switzerland). All data was acquired and processed using VisionCATS 2.1 software (Camag, Muttenz, Switzerland).

**FIGURE 1:**
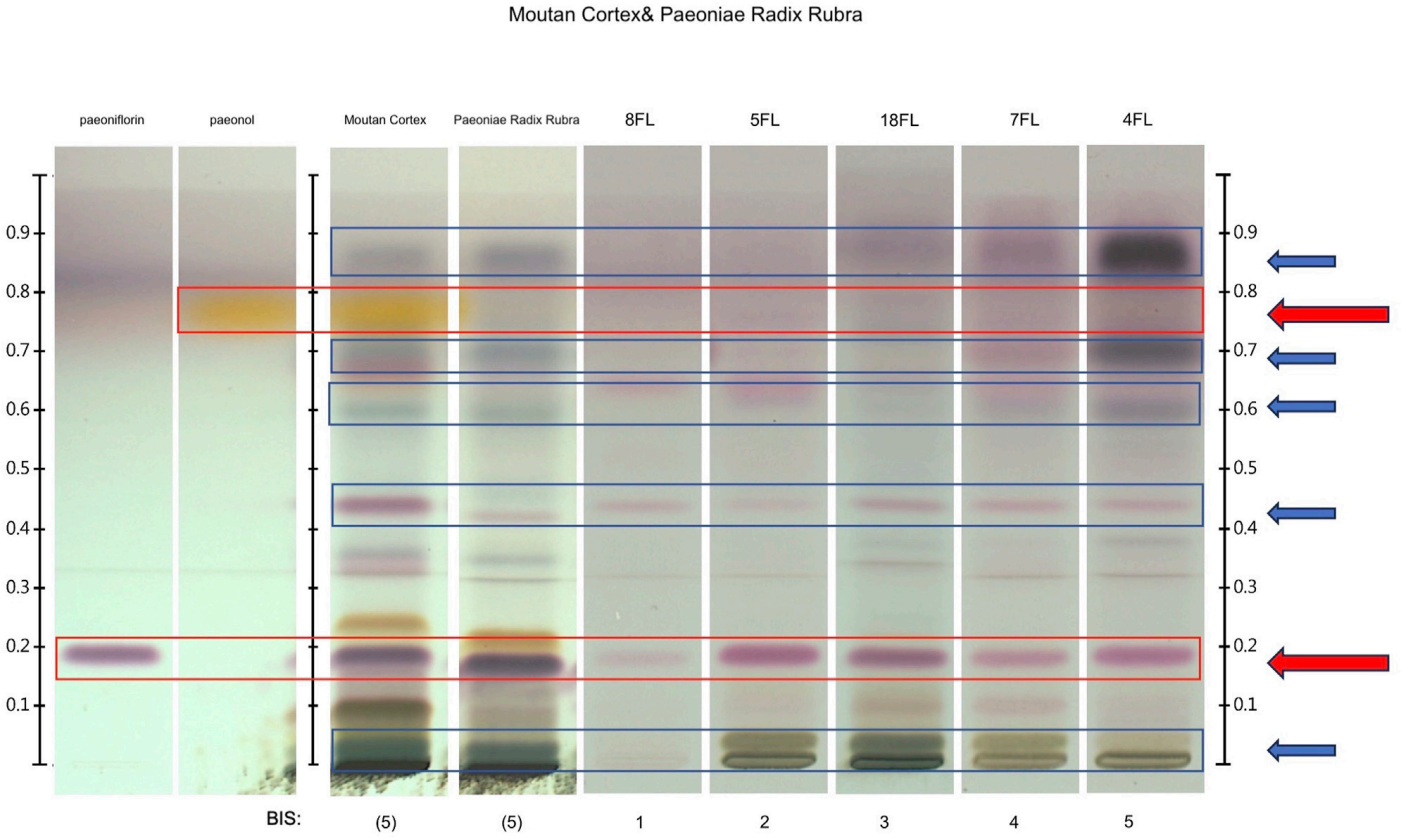
Example for the evaluation of Band Intensity Score (BIS) with scores 1-5 for formula FL with the ingredients of Moutan Cortex and Paeoniae Radix Rubra. Pharmacopoeial reference standards used are paeoniflorin (expected both in Moutan Cortex and Paeoniae Radix Rubra), paeonol (expected only in Moutan Cortex), and botanical references for each Moutan Cortex and Paeoniae Radix Rubra. Red arrows and frames highlight the bands corresponding to pharmacopoeial reference standards for Moutan Cortex and Paeoniae Radix Rubra. Here, paeonol is visible for Moutan Cortex, while all the multi-ingredient samples do not show this band. Paeoniflorin, the other reference standard, is visible in all samples, but with different intensities. Blue arrows and frames highlight the band patterns of the botanical references, which are the basis for a more representative fingerprint (“holistic”) and create, together with the reference standards, the fundament for the evaluation of BISs.

For chemical fingerprints, all ingredients are named with herbal drug names. Each sample was compared to the fingerprint of each single expected ingredient (the Pharmacopoeial botanical standard). To display the results, a Band Intensity Score (BIS) with a scale from zero to five was visually assigned for all ingredients of samples for formulae FL and GX. Each band in the fingerprints was given a score from zero to five based on the intensity perceived by the naked eye, compared to the standard, where zero is “not detectable”, and five is the highest intensity. The combination of these scores for one ingredient, was summarized by assigning a Band Intensity Score (BIS) to each ingredient in every sample (see Figure 1 as an example). BISs with scales 1-5 refer to the quality of the ingredients’ entire fingerprint with respect to the visibility and positions of bands for the pharmacopoeial reference marker compounds and botanical references in each chromatogram. See Supplementary Figures S1, S2 for ingredients in the FL and GX formula, respectively.

Pharmacopoeial reference standards used are paeoniflorin (expected both in Moutan Cortex and Paeoniae Radix Rubra), paeonol (expected only in Moutan Cortex), and botanical references for each Moutan Cortex and Paeoniae Radix Rubra. Red arrows and frames highlight the bands corresponding to pharmacopoeial reference standards for Moutan Cortex and Paeoniae Radix Rubra. Here, paeonol is visible for Moutan Cortex, while all the multi-ingredient samples do not show this band. Paeoniflorin, the other reference standard, is visible in all samples, but with different intensities. Blue arrows and frames highlight the band patterns of the botanical references, which are the basis for a more representative fingerprint (“holistic”) and create, together with the reference standards, the fundament for the evaluation of BISs.

## Results

According to their classical formulations, the formula Gui Zhi Fu Ling Wan (FL) incorporates five ingredients, while the formula Ge Xia Zhu Yu Tang (GX) contains 12 ingredients, of which one of animal origin. The five ingredients of FL correspond to six plant species and one fungal species, *Cinnamomum cassia* (L.) J. Presl, *Paeonia lactiflora Pall.*, *Paeonia suffruticosa Andrews*, *Paeonia veitchii* Lynch, *Prunus davidiana Franch*, *Prunus persica* (L.) Batsch and *Poria cocos* (Schw.) Wolf. The 12 ingredients of GX correspond to 15 plant species. The plant species are *Angelica sinensis* (Oliv.) Diels, *Carthamus tinctorius* L., *Citrus aurantium* L., *Corydalis yanhusuo* (Y.H.Chou & Chun C.Hsu) W.T.Wang ex Z.Y.Su & C.Y.Wu, *Cyperus rotundus L.*, *Glycyrrhiza glabra L.*, *Glycyrrhiza inflata Batalin*, *Glycyrrhiza uralensis Fisch.*, *Lindera aggregata* (Sims) Kosterm., *Ligusticum striatum DC.*, *Prunus davidiana Franch.*, *Prunus persica* (L.) Batsch, *Paeonia lactiflora Pall.*, *Paeonia suffruticosa* Andrews, *Paeonia veitchii* Lynch, Species for *Prunus* and *Paeonia* can each be sourced from two plant species, and species for *Glycyrrhiza* can be sourced from three plant species. For each formula, the specific order of herbs according to formulation techniques in TCM (Duan et al., 2018) is described in Supplementary Table S3.

In total, 39 possible substitutes were recorded as putative ingredients for the expected species (see Supplementary Table S1). For example, *A. sinensis* has seven unofficial substitutes. The foremost are *A. acutiloba* (Siebold & Zucc.) Kitag., which is also categorized as an alternate species or local variant (Scheid et al., 2015), and *Levisticum officinale W.D.J.Koch*. Lesser known substitutes, which are primarily traded locally in China, are *Angelica megaphylla* Diels, *Angelica gigas* Nakai, *Angelica polymorpha* Maxim., *Ligusticum glaucescens Franch.*, and *Hansenia forbesii* (H.Boissieu) Pimenov & Kljuykov (Bensky et al., 2004; Leon and Yu;Lin, 2017). Generally, Paeoniae Radix Rubra (Chi Shao) and Paeoniae Radix Alba (Bai Shao) are differentiated in the ingredient lists (Supplementary Table S2). In all classical texts, their properties are discussed under the single heading of Paeoniae Radix (Shao Yao), though it is recognized that the red and white inflorescences are very distinct in their therapeutic action (Scheid et al., 2015).

Sample 2GX includes *Nelumbo nucifera* Gaertn. (Lian Fang) in the ingredient list. For samples 3GX and 4GX, the resins of frankincense, *Boswellia carterii* Birdw. (Ru Xiang) and myrrh, *Cammiphora myrrha* Engl. or *Balsamodendrum ehrenbergianum* O. Berg (Mo Yao) are included in the ingredient list. Here, frankincense is used as an additional ingredient deviating from the classical formulae as it is common in the treatment of endometriosis (Cho et al., 2023). Sample 1GX and 5GX include the ingredient Trogopterori Faeces (Wu Ling Zhi), a product of animal origin. Other ingredients are listed for 4GX, such as activated carbon, botanical wax, and talcum, and for 8 FL fillers and binders of corn starch (non-GMO), dextrin, activated carbon, and gelatin (pork).

### Dual loci metabarcoding for TCM authentication

**FIGURE 2:**
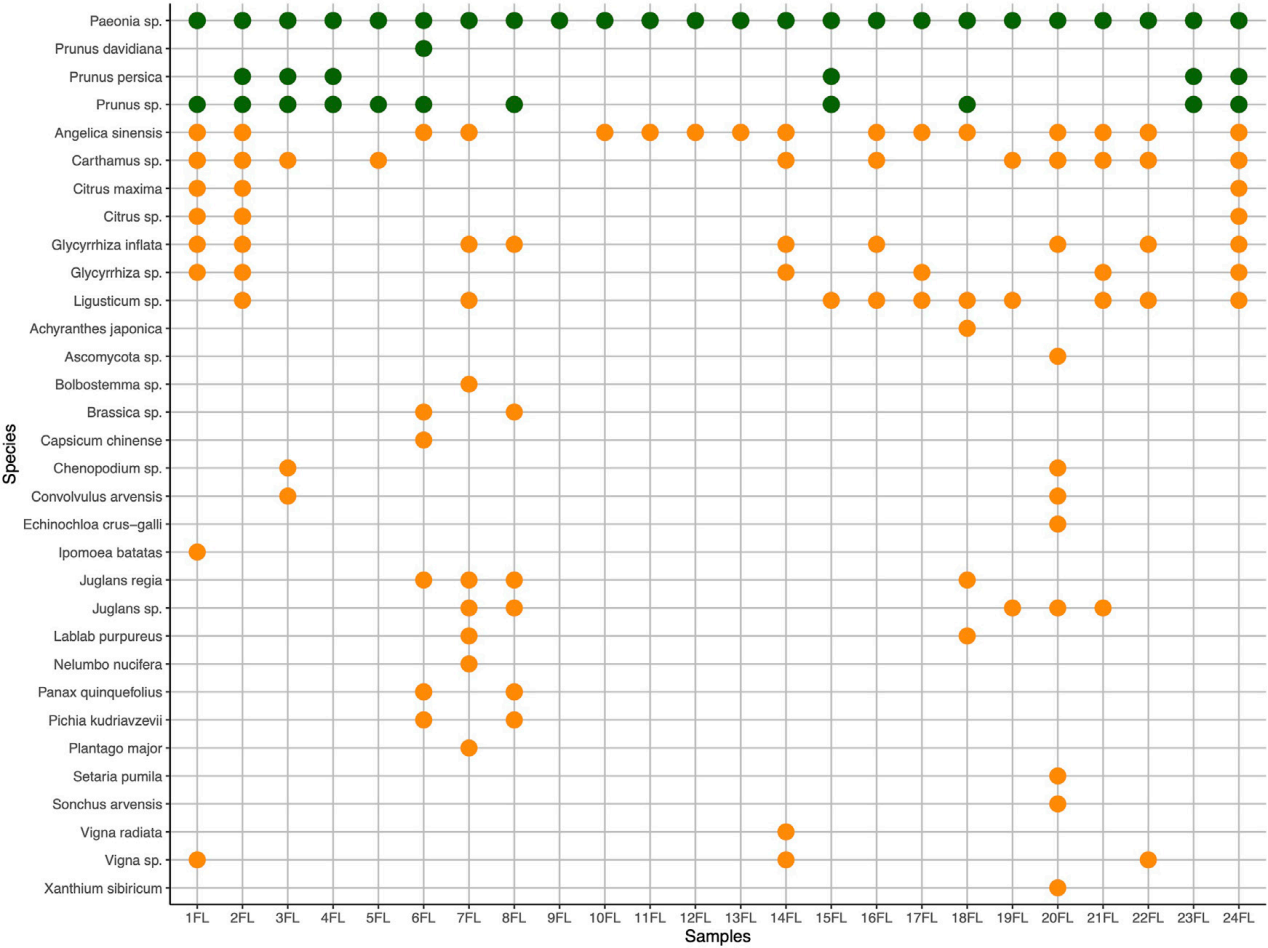
Expected (green) and unexpected (orange) species authenticated in products and mixtures of formula Gui Zhi Fu Ling Wan (FL) using DNA metabarcoding. Undetected expected ingredients Cinnamomi Ramulus (*Neolitsea cassia* (L.) Kosterm., syn. *Cinnamomum cassia* Presl.) and Poria Cocos (*Poria cocos* (Schw.) Wolf), have zero identification hits and are not reported in the graphics. *Ad hoc* mixtures (AM) include samples 1-3FL, 23FL, and 24FL.

**FIGURE 3:**
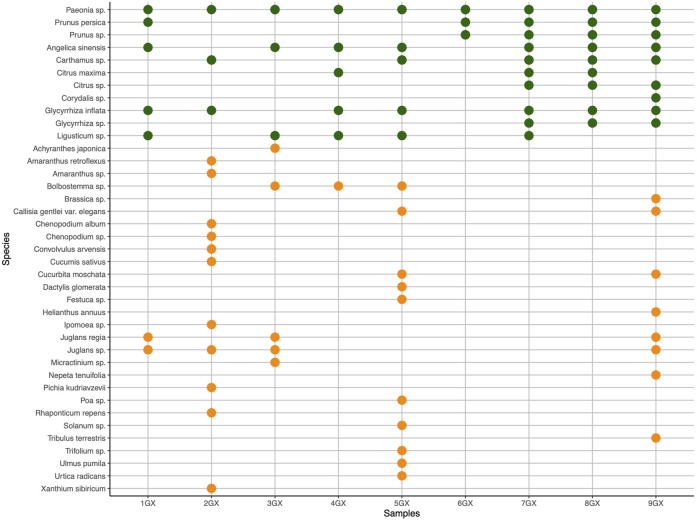
Expected (green) and unexpected (orange) species authenticated in products and mixtures of formula Ge Xia Zhu Yu Tang (GX) using DNA metabarcoding. Undetected expected ingredients: Linderae Radix (*Lindera aggregata* (Sims) Kosterm), Cyperi Rhizoma (*Cyperus rotundus* L.), with zero identification hits and are not reported in the graphics. *Ad hoc* mixtures (AM) include samples 7-9GX.

A dataset consisting of 1 620 141 reads was obtained for nrITS1 with an average of 49 095 reads per sample. Respectively, a dataset with 1 647 249 reads was obtained for nrITS2 with an average of 49 917 reads per sample. Operational taxonomic units (OTUs) were obtained for all sample mixtures for markers nrITS1 and nrITS2. The raw dataset of nrITS1 contained 361 OTUs, and after applying strict quality selection criteria and pooling together OTUs assigned to similar species, 62 unique species were obtained. The raw dataset of nrITS2 contained 291 OTUs, whilst after applying the quality criteria 34 species were obtained. All sample preparations had OTUs that passed the bioinformatic trimming and filtering criteria and all samples could be included in the results. A complete overview of the detected species per sample mixture and the corresponding number of reads is provided in Supplementary File S1. After applying a threshold for relative species abundance greater than 0.002 to the identified taxa (Yao et al., 2022), a total of 30 ingredients were identified on the species level and 18 ingredients on the genus level for both primers, ITS1 and ITS2. A separate analysis for ITS1 resulted in a total of 24 plant ingredients on the species level and a total of 16 plant ingredients on the genus level with one extra ingredient belonging to the phylum of Ascomycota. A total of 12 plant ingredients were detected at the species level, and 6 ingredients at the genus level for nrITS2, except for one ingredient identified as green algae, *Micractinium* sp. The number of ingredients detected per sample ranged from 1 to 16. A total of 22.4% of the identified species were expected ingredients. Assessing the sources of all unexpected ingredients, we found that 39.5% were crops, such as mung bean and sweet potato, 21.1% were weeds, while another 5.3% may be weeds or crops such as clover or animal feed, 5% were types of grass, while 10.5% are made up of trees, green freshwater algae, fungi and ornamentals. Another 13.2% of ingredients are medicinal botanicals and find use in various traditional East Asian medicinal systems. Amongst those plant species that commonly find use in TCM, *Xanthium sibiricum* Patr. (Pinyin: Can Er) was detected with nrITS1 in two samples, 20FL (see Figure 2) and 2GX (See Figure 3) (Supplementary Table S2).

The results for the authentication of samples from each formula, FL and GX, and barcode ITS1 and ITS2, are discussed separately. For FL, analysis with barcode nrITS1 detected only three potential expected ingredients. *Prunus* species are present, and in most cases specifically associated with the detection of *Prunus persica* L. Batsch and *P. davidiana* Franch. (Pinyin: Tao ren) (both species are accepted as sources for Persicae Semen) (see Supplementary Figure S3A). The detection of *Paeonia* species could account for either or both Paeoniae Radix, which is equivalent to the scientific names *Paeonia lactiflora* Pall. or *P. ostii* T.Hing & J.X.Zhang (Pinyin: Shao yao), and Moutan Cortex, which is *P. suffruticosa* Andrews as both ingredients are sourced from *Paeonia* species, but consequently this results in the impossibility to distinguish between the two ingredients. The two ingredients, Cinnamomi Ramulus (Gui zhi), associated with *Cinnamomum cassia* (L.) J.Presl and other possible substitutes, and Poria cocos, sourced uniquely from *Poria cocos* (Schw.) Wolf, could not be detected across the entire sample range including the *ad hoc* preparations AM1 FL (1-3FL) and AM2 FL (23, 24FL). They are therefore not reported in Supplementary Figure S3A). On the other hand, AM1 FL (1-3FL) and 24FL seem to include plant species from formula GX. Furthermore, Angelica sinensis Radix (Dang gui), sourced uniquely from *Angelica sinensis,* is present in 15 samples. It is a prominent ingredient in herbal formulations for women’s health. Two samples, 19 and 20 FL, did not show any expected ingredients, again these are tablets purchased online (See Supplementary Table S2).

For FL, analysis using ITS2 barcode managed to identify only two expected ingredients, namely ingredients of the genus *Prunus* and *Paeonia* (see Supplementary Figure S3B). The genus *Paeonia* is found across the whole sample range. AM1 FL (1, 2FL) and 24FL samples seem to include plant species belonging to formula GX, like *A. sinensis* and *Glycyrrhiza inflata* Batalin (Gan cao), which are prominent TCM herbs.

When using nrITS1 on GX samples, seven out of 11 expected ingredients were identified across the sample range (see Supplementary Figures S3C). Samples 1, 3, 4, and 6 GX have only three ingredient hits. Among the analyses of the *ad hoc* preparations, AM 9 GX differs from the other two samples for ingredient abundance. The only expected ingredient, which was possible to identify at the species level, was *A. sinensis*. Sample 5 GX shows several outliers and unexpected ingredients.

Using barcode nrITS2 for the GX formula, it was possible to identify six out of 11 expected ingredients across the sample range (see Supplementary Figures S3D); notably, the presence of *Ligusticum* species could not be detected, as seen with ITS1. The *ad hoc* preparations (7-9GX) show similar scores to each other for the expected ingredient’s identity and abundance. The genus of *Juglans* and *Bolbostemma* are found abundantly across the sample range. *A. sinensis*, *G. inflata,* and *Citrus maxima*, the latter most likely a substitute species for *C. aurantium* (Zhi ke) were identified at the species level.

### HPTLC

The band positions and visibility of the chemical markers of the single ingredients in the FL and GX formulas appear with characteristic colors and Rf values. All botanical reference materials show clear chromatograms and all marker compounds were identified (Supplementary Figures S1, S2).

With HPTLC, we obtained 130 positive hits for expected ingredients in formula Fl with 20 samples and GX with seven samples, including the AMs. Overall, a total of 70,7% of expected ingredients were identified across the sample range. Single ingredients refer to the accepted species under the ingredients as listed in the Chinese Pharmacopoeia (Chinese Pharmacopoeia Committee, 2020). Moutan Cortex could not be identified with certainty. In the FL samples, the band of paeonol, typical for Moutan Cortex, is not visible in any of the samples. On the other hand, the paeoniflorin band which is typical for both Paeoniae Radix Rubra and Moutan Cortex can be detected in the majority of the samples. This result does not rule out the presence of either, but it means that the sample contains at least one of those two ingredients which is further explored in this discussion.

**FIGURE 4:**
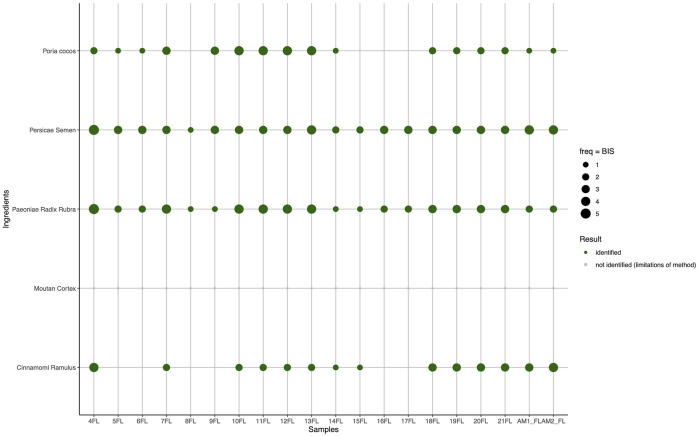
HPTLC detection of target ingredients in products and mixtures of formula Gui Zhi Fu Ling Wan (FL) based on BISs. The freq (frequency) of the dots represents the numerical score assigned (0 absent, 1 faintest, 5 most intense). The BIS is directly correlated to the identification success rate of the individual ingredients in the samples. Hence, the green dot size visualizes the scoring level of identification for target ingredients, as described in the method section. Moutan Cortex (gray) could not be determined due to the limitations of the method.

Among the FL formulae (Figure 4), Cinnamomi Ramulus is the least detected ingredient, showing only in 14 out of 20 samples, followed by Poria cocos, in 16 out of 20 samples. The rest of the sample fingerprints show the presence of all expected ingredients, while Moutan Cortex and Paeoniae Radix Rubra could not be unambiguously distinguished since the paeonol band of Moutan Cortex was not detectable.

**FIGURE 5:**
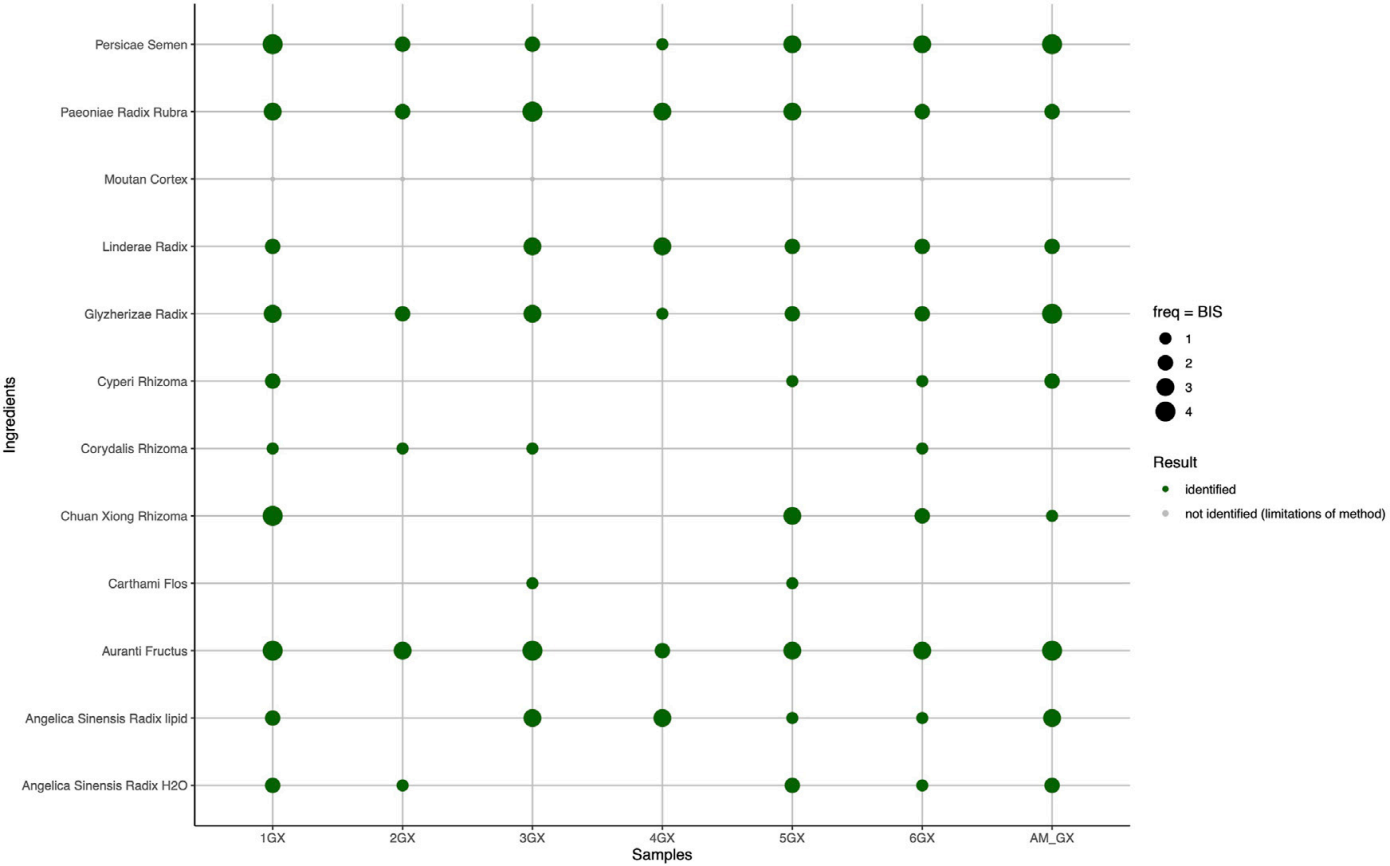
HPTLC detection of target ingredients in products and mixtures of formula Ge Xia Zhu Yu Tang (GX) based on BISs.

In the GX formula samples (Figure 5), Corydalis Rhizoma and Carthami Flos in addition to Moutan Cortex were not identified in the *ad hoc* preparation AM GX, even though the ingredients mixed in these preparations, when analyzed individually, could be clearly identified (see Supplementary Figure S4.) Samples 2GX and 4GX were deemed to be the samples with the poorest ingredient composition, with three undetected ingredients, namely Cyperi Rhizoma, Chuan Xiong, and Carthami Flos. Carthami Flos went undetected in five of seven samples and was detected only with faint bands (BIS = 1) in the last two.

## Discussion

Several studies have highlighted the potential of multi-locus DNA barcoding for the identification of TCM ingredients. The biggest advantage of DNA metabarcoding is to simultaneously identify numerous species in complex and processed herbal preparations (Zhu et al., 2022). Nonetheless, the limitations of DNA metabarcoding are framed by the quality, processing state, or product type of extracted material, the DNA purification procedure, choice of primers, markers, amplification protocols for library preparation, sequencing method, bioinformatic filtering and setting for qualitative and clustering thresholds (Raclariu-Manolică et al., 2023a). The accuracy of DNA metabarcoding further requires a comprehensive taxonomic reference library (Taberlet et al., 2007). Shortcomings of barcoding exist due to incomplete data in reference databases for barcodes (Zhu et al., 2022) and challenges with delimitating species with species delimitation models (Taylor and Harris, 2012; Howard et al., 2020; Puillandre et al., 2021). Chemical fingerprinting then complements approaches focused on biological characteristics, i.e., DNA, morphology, etc. Chemical analysis allows us to distinguish between the different parts of the plant used, e.g., the root as opposed to the leaves; this is a non-trivial point, as adulteration can also occur by means of using other (generally cheaper) parts of the plant that are not the ones required for the desired medicinal effect (Ichim and de Boer, 2020). Furthermore, chemical fingerprinting methods enable the assessment of a full qualitative profile, as well as the identification of low-quality aspects of the phytochemical contents, and the detection of significant product-to-product variation (Ichim and Booker, 2021). High-perfomance thin-layer chromatography (HPTLC) is the most advanced form of thin-layer chromatography (TLC) and a robust method for quality control of single,- and poly-herbal formulations (Velho;Pereira et al., 2011; Nicoletti et al., 2013; Booker et al., 2014; Booker et al., 2016; Nandanwadkar et al., 2016; Kandil et al., 2022) and pharmaceutical drugs (Shewiyo et al., 2012). It can be used for the analysis of multiple samples simultaneously and efficiently at a relatively low price (Kunle, 2012; Sonia, 2017; Kandil et al., 2022). Its advantages are improved resolution, detection sensitivity, and enhanced *in situ* densitometric quantification compared to ordinary TLC (Sonia, 2017).

The combination of the internal transcribed spacers regions, nrITS1, and nrITS2 provided the most comprehensive species identification for two short barcode markers as the most sequenced regions for molecular analysis of TCMs at lower taxa levels (Chen et al., 2017; Yao et al., 2022; Zhu et al., 2022). The results from nrITS1 and nrITS2 can be used in an integrative approach to receive an enhanced informative overview of the ingredient profile (Zhu et al., 2022) of samples from FL and GX. NrITS1 and nrITS2 differ in taxonomic identification at species and genus levels. Here, nrITS1 performed generally better in terms of the number of OTUs with taxonomic assignment. However, neither the nrITS1 nor nrITS2 data yielded OTUs for all expected genera in the herbal formulae FL and GX. For example, the presence of Cinnamomi Ramulus (Pinyin: Gui zhi) was confirmed with the HPTLC analysis, but *Neolitsea cassia* (L.) Kosterm (syn. *Cinnamomum cassia* (L.) J.Presl) could not be detected for any of the FL samples. This discrepancy is typical for the nature of TCMs as highly processed herbal remedies. *Pao Zhi*, the traditional processing technique, and other processing methods in TCM are likely to affect the DNA quality through measures like cutting, crushing, roasting, baking, stir-frying, and the application of liquid or solid excipients (Engelhardt et al., 2018; Wu et al., 2018). Furthermore, a methodological challenge for DNA-based identifications in TCMs is that plant compounds including polysaccharides, polyphenols, lipids, essential oils, and alkaloids, can interfere with DNA extraction and PCR amplification. Interference from those compounds or initial processing techniques of source material can yield false-negative results. As such, dominant ingredients with relatively intact DNA compared to other ingredients may yield amplification biases and in turn skew the sequencing results towards those ingredients. For DNA extraction from TCMs and complex, poly-herbal samples it is recommended to employ tailored extraction protocols (Corrado, 2016; Lo and Shaw, 2019). Looking at the *ad hoc* mixtures for FL, where input material for each ingredient was previously thoroughly mixed in equal proportions, we may reveal a common issue of homogeneity for the mixing of different powdered herbal material for consecutive DNA extraction from smaller amounts of the total mix, where some plant ingredients might be overrepresented. Respectively, homogeneity might be an issue in the manufacturing processes of Chinese herbal products, where grand amounts of mixed herbal materials are compressed in small amounts into tablets or capsules. This may have furthermore led to potential false-negative detection of species present at low abundance in the samples. We may also observe false-positive reads from micro contaminations of other species which are common in non-sterile herbal manufacturing processes (weeds, etc. during harvesting, packaging, and handling). These types of contamination, in minute quantities, are common in pharmacy preparation rooms and do not usually affect the quality, safety, and efficacy of these types of preparations. It should be noted that the monographs on “herbal drugs” of the European Pharmacopoeia allow for up to 2% of foreign matter unless differently stated in a specific herb monograph (EMA, 2006; EMA, 2011). Despite this, in the case of sample 5GX, we have Trogopterori Faeces included in the ingredients list of the product, which may be the reason for ingredient outliers detected with nrITS1. Nonetheless, the analysis detected some use of suspected unreported fillers as well as suspected adulteration. In two samples, in both FL and GX, we detected *Xanthium sibiricum* Patr. This species is toxic if bigger amounts are ingested and can cause serious health risks (Gurley et al., 2010; West et al., 2010; Scheid et al., 2015). Another species detection of interest is *Nelumbo nucifera* Gaertn. in 7FL and 2GX. Here we suspect a label switch for the ingredient. 7FL analyzed contains *Nelumbo nucifera* Gaertn., which is only expected for sample 2GX. Both samples originate from the same company and were custom-made products (See Supplementary Table S2). Following these assumptions, we can notice problems with nomenclature, and correct labeling on the market and we have reasons to assume fraudulent practice for two of the samples.

The HPTLC method presented provides qualitative estimates of the ingredient profiles of each sample, yielding a useful insight into the quality of herbal mixtures. The whole chemical profile of each plant species was considered for the two formulae FL and GX extracting a common pattern as a criterion for elaborating the individual formulations (Noviana et al., 2022). The multi-ingredient chromatographic profiling attempts to separate the individual components and to develop a fingerprint for each sample, which allows for a qualitative evaluation, similar to Nicoletti et al. (2013). Fingerprints of the marketed multi-ingredient botanicals were compared with fingerprints of constituent extracts for each single ingredient and the fingerprint of a respective reference botanical material. The presence of each ingredient in the formulae could be analyzed against their botanical references and their pharmacopeial marker(s). Three *ad hoc* mixtures were prepared and partly served for validation of the method. The *ad hoc* mixtures, AMs, were prepared using single ingredients that were individually tested with HPTLC that successfully confirmed their identity (see Supplementary Figures S4A–M). To compare results for different samples, each fingerprint needed to be quantified. A scoring scheme, the Band Intensity Score (BIS), was further developed for this purpose. Ideally, one would aim for an unequivocal numerical score, but when dealing with multiple ingredients containing multiple chemical constituents, the analysis becomes more complex. The basic points for evaluation remain those typical of HPTLC, which are the presence or absence of desired bands, and their intensity. Therefore, we evaluated each ingredient, first according to the presence of chemical marker compounds, and then for their proximity to other elements present in the botanical references’ fingerprints. The method cannot solely rely on the presence or intensity of the marker compound, but needs to be combined with a holistic evaluation of the total fingerprint of an ingredient. Typically, BIS assigns a numerical value to the comparative intensity of bands, but for instance, in the case of Paeoniae Radix Rubra (Figure 1), sample 7FL scored higher than sample 18FL, even though the band for the chemical marker compound is less intense. Looking at the entire fingerprint of the ingredient, in comparison with the botanical reference, is more accurate here. The evaluation of BIS requires an experienced eye, and remains susceptible to subjective interpretation, even though positive or negative detection is quite unequivocal. Generally, we face methodological challenges when analyzing multi-herbal formulae, such as complex TCM preparations with HPTLC and unilateral standardization of methods is not possible. The initial quantity of single ingredients within a preparation can vary, which ultimately leads to a variation of the concentration of chemical marker compounds and other chemical compounds which consequently alters the fingerprint. When the number of ingredients increases, the concentration of each ingredient and its components will consequently be reduced. HPTLC will therefore meet challenges when there are numerous ingredients, as for the GX formula with 12 ingredients. Three ingredients were not detectable in the AM_GX, while signals from only one marker compound were absent for AM_FL. Various chemical compounds of the multi-herbal complex can cause chemical reactions and change or overlay the expression of certain bands. We see this in the case of the band for chemical marker compound paeonol for the detection of Moutan Cortex, which is not visible in the fingerprint for any of the multi-ingredient samples, even though we have a proof of its presence in the single ingredient analysis of the *ad hoc* preparations (see Supplementary Figures S4B). Besides, concentrations for chemical marker compounds can vary for botanical materials of varying origins amongst different plant ingredients and amongst the same ingredients, as well. For instance, paeonol is represented in lower concentrations with 0.9%–2.2% in ingredient Moutan Cortex (He et al., 2006; Tobyn et al., 2011), which is plant species *Paeonia suffruticosa Andrews*, than, for example paeoniflorin with 4.6%–5.1% in ingredient Paeoniae Radix Rubra, which can be plant species *Paeonia anomala* subsp. veitchii (Lynch) D.Y.Hong & K.Y.Pan or *Paeonia lactiflora Pall.* (Cai et al., 1994). Furthermore, it is important to consider natural fluctuations in chemical compounds for different growth cycles, eco-regions, and times of the year. E.g. for *P. lactiflora*, paeoniflorin content was found to be highest in November (Yamamoto, 1988). Some accepted TCM ingredients can be sourced from varying plant species, whilst the standard identification method in the pharmacopeia remains the same for either species. Hence slight differences can be expected for the fingerprints of various samples. According to TCM processing techniques, plant material can further be sourced from differently treated botanical materials (Wang et al., 2019) (fried, boiled, etc.), which can alter the chemistry of chemical compounds. Additionally, In TCM practice, the ratios of different ingredients within a formulation can vary according to individual TCM diagnostics. These factors combined may result in diverse chemical fingerprints of seemingly identical TCM preparations.

Different types of processing techniques have different effects on the content of chemical constituents. For instance, concentrations of paeoniflorin are lower in boiled *Paeonia lactiflora* Pall. due to thermal instability (Cai et al., 1994). From a chemical analytical point of view, we suggest preparing unique standards for each of the accepted ingredients and ultimately multi-herbal formulae *ad hoc*. In practice, for instance, for the ingredient Paeoniae Radix Rubra, this would mean firstly to prepare first standards from vouchered botanical material of both accepted species listed under the ingredient according to the Chinese Pharmacopoeia. Secondly preparing standards from the three possible TCM processing techniques, which are dry-frying, wine-frying, or vinegar-frying of dried slices from the root (Scheid et al., 2015). And thirdly establishing a dictionary for all different fingerprints, which are multi-fold for all possible ingredients and processing techniques of single ingredients within a formula. Each sample representing a polyherbal formula would then have to be compared to the full spectrum of varying fingerprints in this dictionary. There are some limitations to this method. We have established that, in some cases, both marker compounds and botanical references are not optimal for the methodological approach, e.g., paeonol for Moutan Cortex. Constraints for the method are the use of multiple, possibly expensive chemical marker compounds, and sometimes difficult to retrieve-botanical reference materials. We do not take into account the sensitivity of the type of raw material and manufacturing processes, and limitations in differentiating between plant species (Bansal et al., 2014). The method has limitations in verifying substitutions and adulterations, especially of highly processed multi-herbal products, as merely the presence or absence of marker and reference compounds can be detected.

## Conclusion

Our study emphasizes that integrating DNA metabarcoding with chromatography gathers and combines a wider set of data to provide a more representative picture of herbal preparations for a more comprehensive authentication of Traditional Chinese Medicines contributing to their quality and safe use. The study presented here, is one of the few combining dual-loci DNA metabarcoding and HPTLC through an integrated approach, to test for the quality and purity of multi-herbal products, in this case of two TCM formulae (FL and GX), commonly prescribed for the treatment of endometriosis. Dual-loci DNA metabarcoding was able to detect two to three out of five expected FL ingredients, and eight to nine out of eleven GX’s expected ones, considering that two ingredients were identified at the same genus level only. In total, 22.4% of the identified species were expected ingredients. Overall, eleven samples of twenty scored higher in identity for FL and six out of nine in GX. Depending on the manufacturing characteristics of each sample, whether they were mixtures *ad hoc*, powders, tablets, granules, capsules, and pill, there are differences in species identification hits. The metabarcoding allowed the identification of 38 unexpected taxa in the sample range. Almost all invested products included other species not listed on the label or known to be an ingredient of the formulae. In contrast, the HPTLC methods were able to detect four out of five expected FL ingredients, and ten out of eleven GX’s expected ones, and revealed a higher fidelity of expected ingredients with 70.7% of expected ingredients.

The study highlighted each method’s drawbacks and strengths, DNA metabarcoding’s sensitivity which allows for the detection of contaminants, and HPTLC’s potential to distinguish between quantities of the plant used. DNA techniques are not bound by the quantity and quality of certain chemical compounds but by the presence of viable DNA which is also dependent on the application of varying TCM processing techniques. This study shows that neither genetic barcoding nor chemical analysis alone, as post-quality-control measures, can trace back the complexity of problems when dealing with poly-herbal products.

## Data Availability

The datasets presented in this study can be found in online repositories. The names of the repository/repositories and accession number(s) can be found below: https://doi.org/10.5281/zenodo.8383538.
