# Supplementary material for: Complementary authentication of Chinese herbal products to treat endometriosis using DNA metabarcoding and HPTLC shows a high level of variability
Source: Front Pharmacol. 2023 Dec 5;14:1305410. doi: 10.3389/fphar.2023.1305410 (PMC10728824; doi:10.3389/fphar.2023.1305410)
Supplement: Supplementary file 1 [file DataSheet1.zip › Table 2.docx]

**Supplementary Table S2. Sample overview of study samples for formula FL and GX**

| Sample_Nr | Accession number  (Source) | Description |
| --- | --- | --- |
| AM1_FL  (1FL+2FL+3FL for genetic samples) | Mix Gui 1 (N&H)  Mix Gui 2 (N&H)  Mix Gui 3 (N&H)  (market) | Grinded Artificial Mixture of Gui Zhi Fu Ling Wan (AM_FL) of crude decoction material from China Town, London.  Composition of 5 ingredients with equal amounts, 1 g for each species from sample set N&H (Gui Zhi, Fu Ling, Mu Dan Pi, Tao Ren, Bai Shao) |
| 4FL | FM19140 (Z)  (pharmacy) | Gui Zhi Fu Ling Wan (Huang Huang), Cinnamomi Ramulus, Paeoniae Rubrae Radix, Poria Sclerotium, Moutan Cortex, Persicae Semen (15 g each)  Powder |
| 5FL | FM19143 (Z)  (pharmacy) | Gui Zhu Fu Ling Wan (10 g each of all 5 ingredients: Cinnamomi Ramulus, Paeoniae Rubrae Radix, Poria Sclerotium, Moutan Cortex, Persicae Semen)  Granules |
| 6FL | FM19144 (Z)  (pharmacy) | Gui Zhu Fu Ling Wan (15 g each of all 5 ingredients: Cinnamomi Ramulus, Paeoniae Rubrae Radix, Poria Sclerotium, Moutan Cortex, Persicae Semen)  Granules |
| 7FL | FM19169 (S)  (European distributor) | Gui Zhu Fu Ling Wan, 499 g, Patient: SGZFLW, Composition of 5 ingredients with equal amounts (Gui Zhi, Fu Ling, Mu Dan Pi, Tao Ren, Bai Shao)  Powder |
| 8FL | FM19171 (GynAs)  (Online market) | Gui Zhu Fu Ling Pian (Cassia twig- Cinnamomum cassia(Gui Zhi), Poria sclerotium- Poria cocos (Fu Ling), Tree peony root bark- Paeonia suffruticosa (Mu Dan Pi), Peach seed- Prunus persica (Tao Ren), Chinese peony root without bark (Paeoniae lactiflora (Bai Shao)) Other ingredients. Corn starch (non_GMO), dextrin, activated carbon, gelkatin(pork). Fillers/binders which together amounts 13% of the tablet weight.  Tablets |
| 9FL | FM19174 (EvSp)  (Online market) | Gui Zhu Fu Ling Wan Tablets gui zhi, fu ling, mu dan pi, chi shao, tao ren |
| 10FL | FM19175 (PC)  (Online market) | Gui Zhi Fu Ling Tan, WG21 Bai Shao Yao, Mu Dan Pi, Tao Ren, Gui Zhi, Fu Ling  Tablets |
| 11FL | FM19176 (PC)  (Online market) | Gui Zhi Fu Ling Tan, WG22 Bai Shao Yao, Mu Dan Pi, Tao Ren, Gui Zhi, Fu Ling  Tablets |
| 12FL | FM19177 (PC)  (Online market) | Gui Zhi Fu Ling Tan, WG23 Bai Shao Yao, Mu Dan Pi, Tao Ren, Gui Zhi, Fu Ling  Tablets |
| 13FL | FM19178 (PC)  (Online market) | Gui Zhi Fu Ling Tan, WG24 Bai Shao Yao, Mu Dan Pi, Tao Ren, Gui Zhi, Fu Ling  Tablets |
| 14FL | FM19179 (N.d.H.)  (Online retailer) | Gui Zhu Fu Ling Wan (1) Ramulus Cinnamomi, Poria, Cortex Moutan, Radix Paeoniae Alba, Persica Semen  Tablets |
| 15FL | FM19180 (N.d.H.)  (Online retailer) | Gui Zhu Fu Ling Wan (2) Ramulus Cinnamomi, Poria, Cortex Moutan, Radix Paeoniae Alba, Persica Semen  Tablets |
| 16FL | FM19182 (H.h.)  (Online retailer) | Gui Zhu Fu Ling Wan (Chi Shao Yao, Mu Dan Pi, Gui Zhi, Fu Ling, Tao Ren)  Tablets |
| 17FL | FM19183 (F009)  (Online market) | Gui Zhu Fu Ling Wan Cinnamon & Hoelen F, F009 Paeoniae Rubrae Radix (Chi Shao Yao), Poria Cocos Sclerotium (Fu Ling), Cinnamomi Ramulus (Gui Zhi), Moutan Radicis Cortex (Mu Dan Pi), Persicae Semen (Tao Ren)  Tablets |
| 18FL | FM19185 (HNHN)  (Online retailer) | Cinnamon & Hoeln Combination, Gui Zhi Fu Ling Wan, Nr.2220 (1) Paeoniae Rubrae Radix (Chi Shao Yao), Poria Cocos Sclerotium (Fu Ling), Cinnamomi Ramulus (Gui Zhi), Moutan Radicis Cortex (Mu Dan Pi), Persicae Semen (Tao Ren) Pills |
| 19FL | FM19187 (HNHN)  (Online retailer) | Cinnamon & Hoeln Combination, Gui Zhi Fu Ling Wan, Nr.2220 (2) Paeoniae Rubrae Radix (Chi Shao Yao), Poria Cocos Sclerotium (Fu Ling), Cinnamomi Ramulus (Gui Zhi), Moutan Radicis Cortex (Mu Dan Pi), Persicae Semen (Tao Ren)  Tablets |
| 20FL | FM19188 (KJ)  (Online market) | Cinnoman and Poria/ Gui Zhi Fu Ling T9 ingredients only in written vernacular Chinese  Tablets |
| 21FL | FM19189 (KJ)  (Online market) | Cinnoman and Poria/ Gui Zhi Fu Ling T10 ingredients only in written vernacular Chinese  Tablets |
| 22FL | FM19192 (J)  (not in HPTLC)  (Online market/ Martin) | Gui Zhi Fu Ling ingredients only in written vernacular Chinese  Tablets |
| AM2_FL  (23FL+24FL for genetic samples*) | Mix Gui 29 (Z)  Mix Gui 30 (Z)  (pharmacy) | Grinded Artificial Mixture ad hoc of Gui Zhi Fu Ling Wan (AM_FL) of crude decoction material Z  Composition of 5 ingredients with equal amounts, 1 g for each species from sample set Z: (Cinnamomi Ramulus, Paeoniae Rubrae Radix, Poria Sclerotium, Moutan Cortex, Persicae Semen) |
| 1GX | FM19141 (Z)  (pharmacy) | Ge Xia Zhu Yu Tang Angelica sinensis Radix, Citri aurantii Fructus, Cyperi Rhizoma, Ligustici Chuanxiong Rhizoma, Moutan Cortex, Persica Semen, Carthami Flores, Corydalis Rhizoma, Glycyrrhiza Radix, Paeoniae rubrae Radix, Trogopterioris Faeces  Granules |
| 2GX | FM19170 (S)  (European distributor) | Ge Xia Zhu Yu Tang, 500g, Patient: SGXZYT: Radix Paeoniae Rubra-Chi Shao, Flos Carthamu- Hong hua, Radix Linderae- Wu Yao, Rhizoma Chuanxiong- CHUAN Xiong, Receptaculum Nelumbinis-Liang Fan, Rhizoma Cyperi- Xiang fu, Radix Angelica Sinenis- Dang Gui, Cortex Moutan- Mu Dan Pi, Rhizoma Corydalis- Yan Hu Suo, Radix Glycyrrhizae Radix- Gan Cao, Semen Persicae- Tao Ren, Fructus Auranti- Chi qiao  Powder |
| 3GX | FM19172 (PF)  (Online retailer) | Ge Xia Zhu Yu San  Ingredients: Angelica sinensis root, Prunus persica seed, Carthamus tinctorius flower, Lindera aggregata root, Ligusticum chuanxiong rhizome, Paeonia suffruticosa root-bark, Paeonia lactiflora root, Cyperus rotundus rhizome, Citrus aurantium fruit-ripe, Boswellia carterii resin, Commiphora myrrha resin, Corydalis yanhusuo rhizome, Glycyrrhiza uralensis root. Pinyin: Dang gui, Tao ren, Hong hua, Wu yao, Chuan xiong, Mu dan pi, Chi shao, Xiang fu, Zhi ke, Ru xiang, Mo yao, Yan hu suo, Gan cao.  Stasis in the Lower Chamber Formula, Ge Xia Zhu Yu Tang  Full Spectrum 5:1 Herb Extract Powder 100% Extract - No fillers or carriers  Extract and crude powder |
| 4GX | FM19173 (PF)  (Online retailer) | Stasis in the lower chamber teapills, Ge Xia Zhu Yu Wan  Ingredients: Angelica sinensis root, Prunus persica seed, Carthamus tinctorius flower, Lindera aggregata root, Ligusticum chuanxiong rhizome, Paeonia suffruticosa root-bark, Paeonia lactiflora root, Cyperus rotundus rhizome, Citrus aurantium fruit-ripe, Boswellia carterii resin, Commiphora myrrha resin, Corydalis yanhusuo rhizome, Glycyrrhiza uralensis root, Activated carbon, Botanical wax, Talcum. - Dang gui, Tao ren, Hong hua, Wu yao, Chuan xiong, Mu dan pi, Chi shao, Xiang fu, Zhi ke, Ru xiang, Mo yao, Yan hu suo, Gan cao.  Pills |
| 5GX | FM19181 (ST)  (European distributor) | Ge Xia Zhu Yu Tang- Persicae & Carthamus Combination 616 B  Ge Xia Zhu Yu Tang, also known as Persica and Carthamus Combination is used for the pattern of Blood stagnation.   \| Name: \| Ge Xia Zhu Yu Tang (blended w/o Wu Ling Zhi) \| \| --- \| --- \| \| Ge Xia Zhu Yu Tang (blended w/o Wu Ling Zhi) is also known as: \| Tangkuei and Corydalis (blended : not cooked) \| \| Manufacturing Process: \| water decocted concentrate(5:1) \|   Ingredients: Peach kernel (tao ren), Carthamus flower (hong hua), Cnidium root (chuan xiong), Tang-kuei root (dang gui), Pteropus (wu ling zhi), Moutan bark (mu dan pi), Red peony root (chi shao), Lindera root (wu yao), Corydalis (yan hu suo), Cyperus root (xiang fu zi), Chih-ko fruit (zhi ke), Chinese licorice root (gan cao)  Capsules |
| 6GX | FM19186 (HNHN)  (Online retailer) | Tangkuei & Corydalis Combination coded with Nr: 3830  Granules |
| AM_GX  (7GX, 8GX,  9GX for genetic samples) | FM3GeXZ  (pharmacy) | Grinded Artificial Mixture ad hoc Ge Xia Zhu Yu Tang (AM_GX) Preparation of standard prescription with plant ingredients from sample set Z, each ingredient according to prescription: 0,6g Dang Gui, 0,6g Chuan Xiong, 0,6g Tao Ren, 0,6g Mu Dan Pi, 0,6g Chi Shao, 0,3g Yan Hu Suo, 0,9g Gan Cao, 0,4,5g Xiang Fu, 0,9g Hong Hua, 0, 4,5g Zhi Ke |

*For DNA metabarcoding we used three technical replicates for all three mixtures prepared ad- hoc, while sample 23 and 24 FL, only have 2 repetitions, since 2 replications were coupled during sequencing.
