# Supplementary material for: Complementary authentication of Chinese herbal products to treat endometriosis using DNA metabarcoding and HPTLC shows a high level of variability
Source: Front Pharmacol. 2023 Dec 5;14:1305410. doi: 10.3389/fphar.2023.1305410 (PMC10728824; doi:10.3389/fphar.2023.1305410)
Supplement: Supplementary file 1 [file DataSheet1.zip › Table 3.docx]

**Supplementary Table S3. Formulae recipes I. FL and II. GX**

1. **Gui Zhi Fu Ling Wan (FL) – Cinnamon Twig and Poria Pill**

Source: Essentials from the Golden Cabinet

Ingredients Dosage recommendation Dosage applied Artificial mixtures

| Cinnamomi Ramulus (gui zhi) | 9-12 g | 1 g |
| --- | --- | --- |
| Poria cocos (fu ling) | 9-12 g | 1 g |
| Paeoniae Radix rubra (chi shao) | 9-15 g | 1 g |
| Moutan Cortex (mu dan pi) | 9-12 g | 1 g |
| Persicae Semen (tao ren) | 9-12 g | 1 g |

Method preparation: In the source text it is advised to grind equal amounts of the ingredient into a powder, form into pills the size of rabbit droppings adding honey and take three times daily before meals. Today, the formula is often prepared as decoction with the dosage as recommended or consumed in forms of pills and taken in 3-6 g doses daily with warm water.

Actions: Invigorates the blood, transforms blood stasis, and reduces fixed abdominal masses.

[(Bensky et al., 2004; Scheid et al., 2015, 2009; Xi and Gong, 2017)](https://sciwheel.com/work/citation?ids=14320539,14320543,9645747,15210719&pre=&pre=&pre=&pre=&suf=&suf=&suf=&suf=&sa=0,0,0,0&dbf=0&dbf=0&dbf=0&dbf=0)

1. **Ge Xia Zhu Yu Tang (GX) – Drive Out Stasis Below the Diaphragm**

Source: Correction of Errors among Physicians (1830)

Ingredients Dosage recommendation Dosage applied Artificial mixtures

| dry-fried Trogopteri Faeces (chao wu ling zhi) | 9 g | 0g |
| --- | --- | --- |
| Angelica Sinensis radix (dang gui) | 9 g | 0.9 g |
| Chuanxiong Rhizoma (chuan xiong) | 6 g | 0.6 g |
| Persica Semen (tao ren) | 9 g | 0.9 g |
| Moutan Cortex (mu dan pi) | 6 g | 0.6 g |
| Paeoniae Radix rubra (chi shao) | 6 g | 0.6 g |
| Linderae Radix (wu yao) | 6-12 g | 0.6 g |
| Corydalis Rhizoma (yan hu suo) | 3 g | 0.3 g |
| Glycyrrhizae Radix (gan cao) | 9 g | 0.9 g |
| Cyperi Rhizoma (xiang fu) | 4.5 g | 0.45 g |
| Carthami Flos (hong hua) | 9 g | 0.9 g |
| Auranti Fructus (zhi ke) | 4.5 g | 0.45 g |

Method preparation: The formula is prepared as a decoction with dosages as recommended.

[(Bensky et al., 2004; Scheid et al., 2015, 2009)](https://sciwheel.com/work/citation?ids=14320539,14320543,15210719&pre=&pre=&pre=&suf=&suf=&suf=&sa=0,0,0&dbf=0&dbf=0&dbf=0)

[Bensky, D., Clavey, S., Stoger, E., 2004. Chinese Herbal Medicine: Materia Medica, Third Edition, 3rd ed. Eastland Pr, Seattle, WA.](https://sciwheel.com/work/bibliography/14320543)

[Scheid, V., Bensky, D., Ellis, A., Barolet, R., 2015. Chinese Herbal Medicine: Formulas & Strategies (Portable 2nd Edition), 2nd ed. Eastland Press.](https://sciwheel.com/work/bibliography/15210719)

[Scheid, V., Bensky, D., Ellis, A., Barolet, R., 2009. Chinese Herbal Medicine: Formulas & Strategies (2nd Ed.), 2nd ed. Eastland Press, Seattle, WA.](https://sciwheel.com/work/bibliography/14320539)

[Xi, S., Gong, Y., 2017. Introduction, in: Essentials of Chinese Materia Medica and Medical Formulas. Elsevier, pp. 417–419.](https://sciwheel.com/work/bibliography/9645747)
