# Supplementary material for: Complementary authentication of Chinese herbal products to treat endometriosis using DNA metabarcoding and HPTLC shows a high level of variability
Source: Front Pharmacol. 2023 Dec 5;14:1305410. doi: 10.3389/fphar.2023.1305410 (PMC10728824; doi:10.3389/fphar.2023.1305410)

Supplementary Figures S1.A. HPTLC Chromatogram of Cinnamomum Ramulus with Band Intensity Scores (BISs)


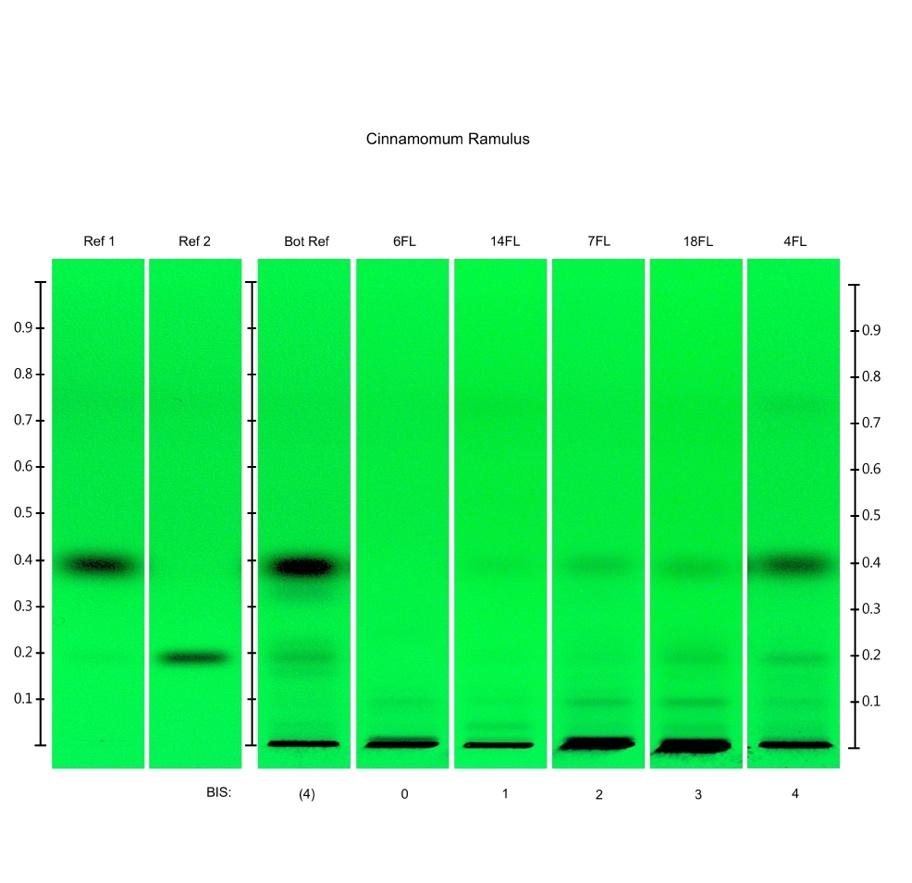


Supplementary Figures S1.B. HPTLC Chromatogram of Poria Cocos with Band Intensity Scores (BISs)


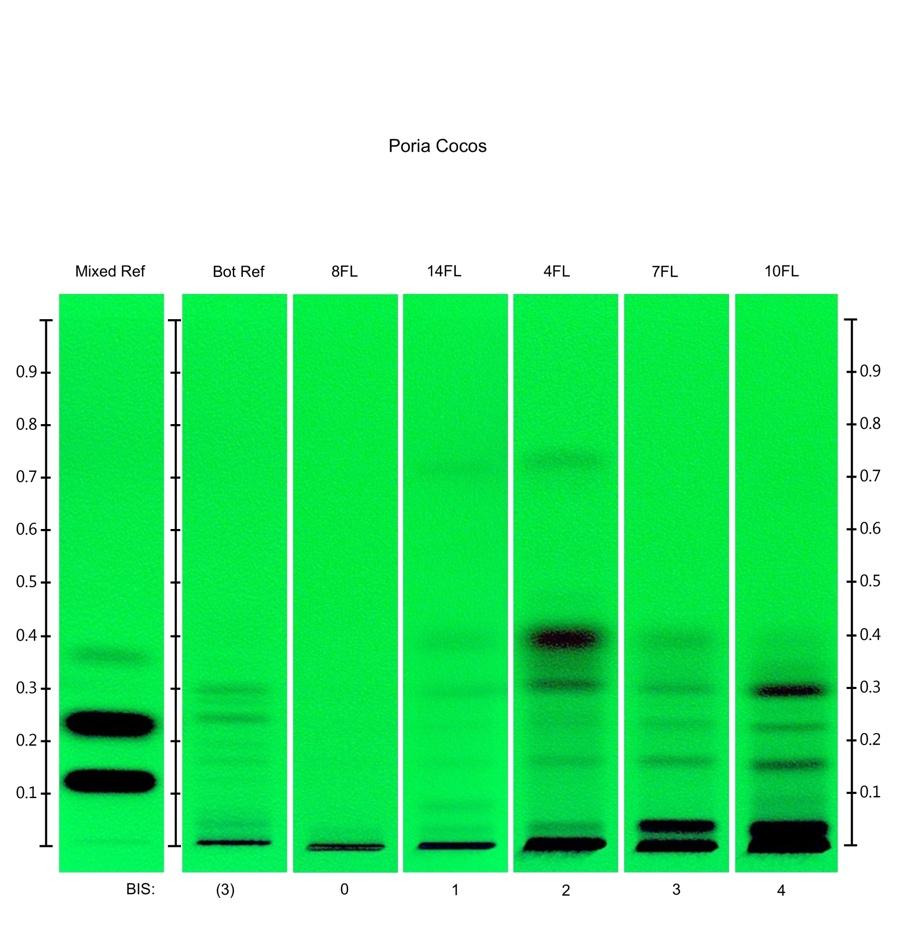


Supplementary Figures S1.C. HPTLC Chromatogram of Paeonia Radix rubra and Moutan Cortex with Band Intensity Scores (BISs)


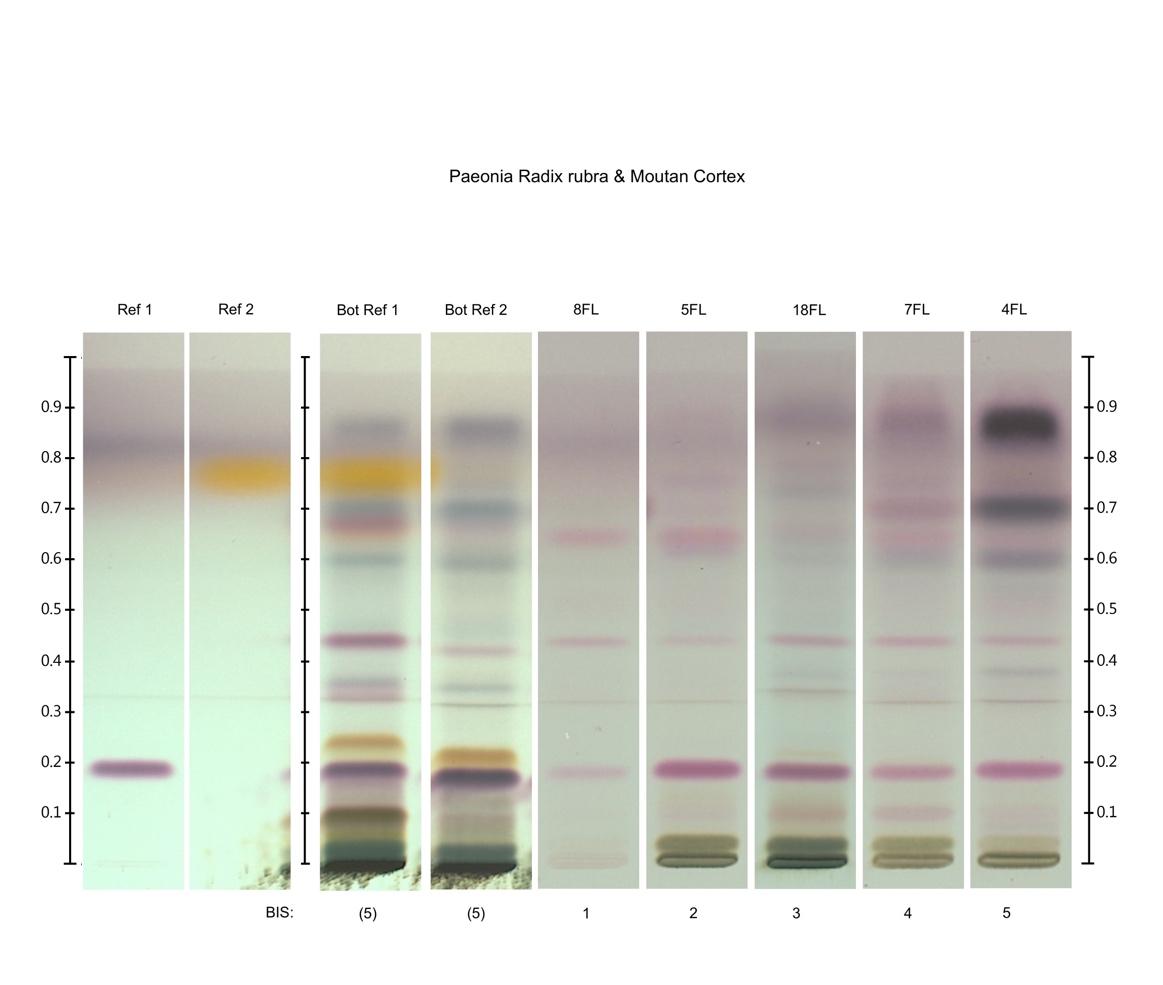


Supplementary Figures S1.D. HPTLC Chromatogram of Persica Semen


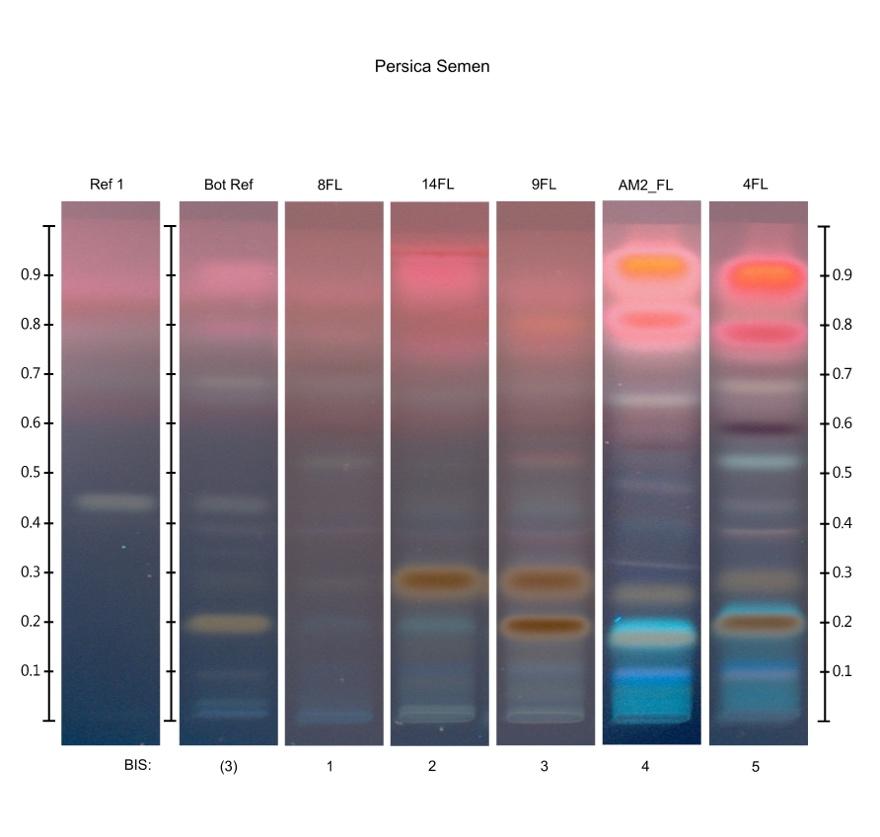

Supplement: Supplementary file 1 [file DataSheet1.zip › Figure 1.docx]
