# Supplementary material for: Complementary authentication of Chinese herbal products to treat endometriosis using DNA metabarcoding and HPTLC shows a high level of variability
Source: Front Pharmacol. 2023 Dec 5;14:1305410. doi: 10.3389/fphar.2023.1305410 (PMC10728824; doi:10.3389/fphar.2023.1305410)

Supplementary Figures S2.A. HPTLC Chromatogram of Angelica Sinensis Lipid Fraction with Band Intensity Scores (BISs)


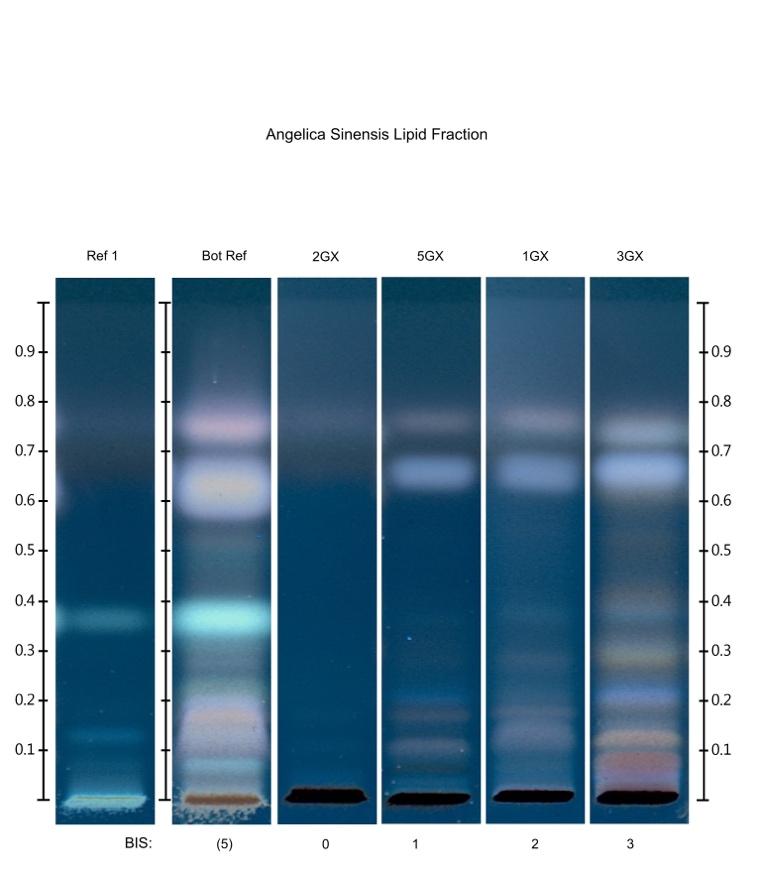

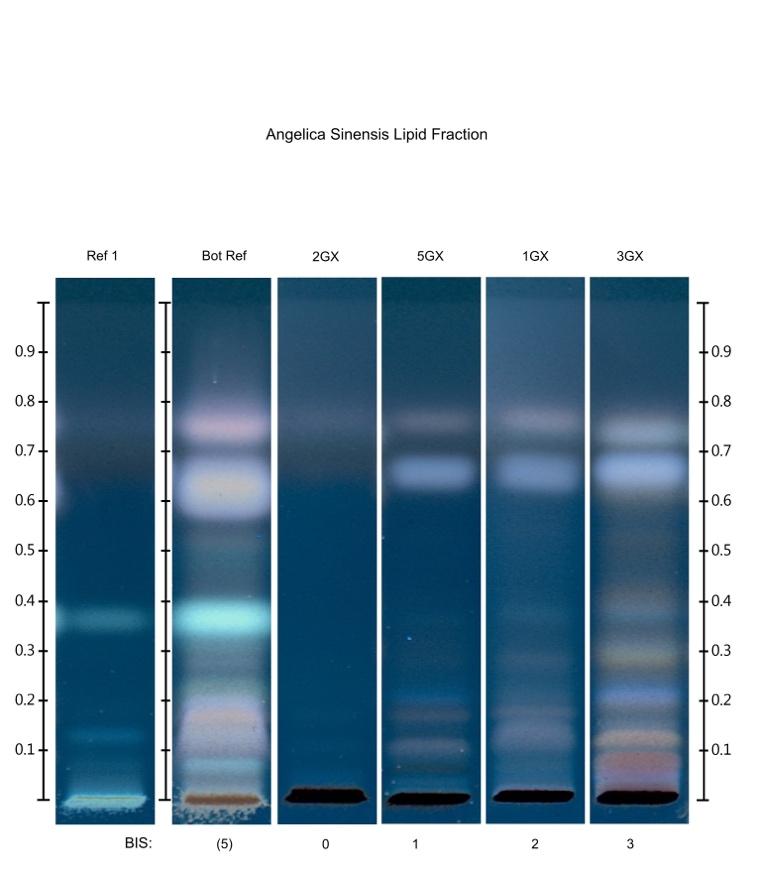


Supplementary Figures S2.B. HPTLC Chromatogram of Angelica Sinensis Water Fraction with Band Intensity Scores (BISs)


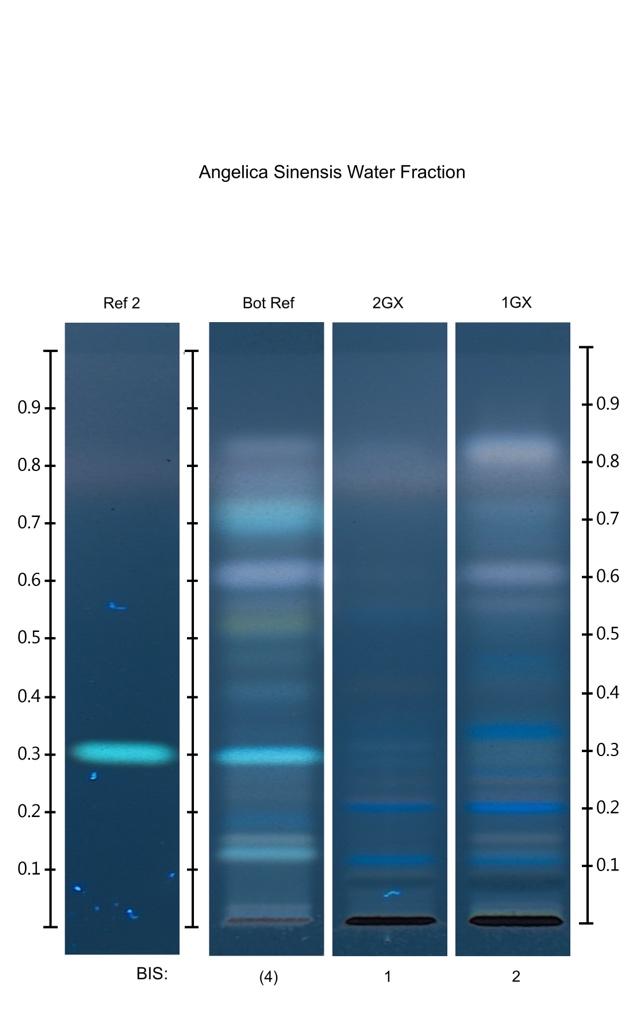


Supplementary Figures S2.C. HPTLC Chromatogram of Persica Semen with Band Intensity Scores (BISs)


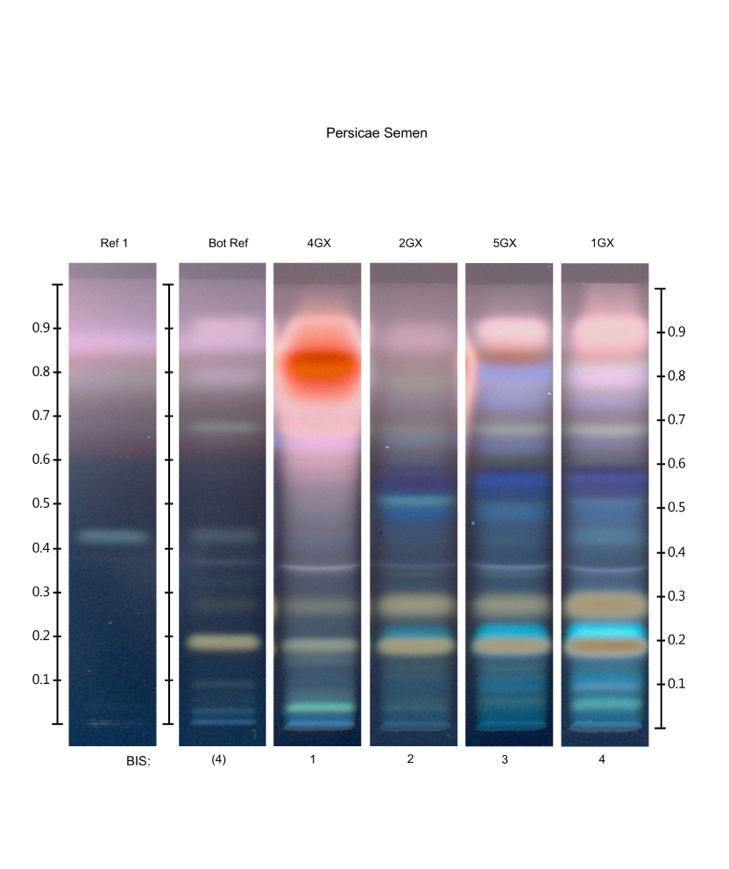


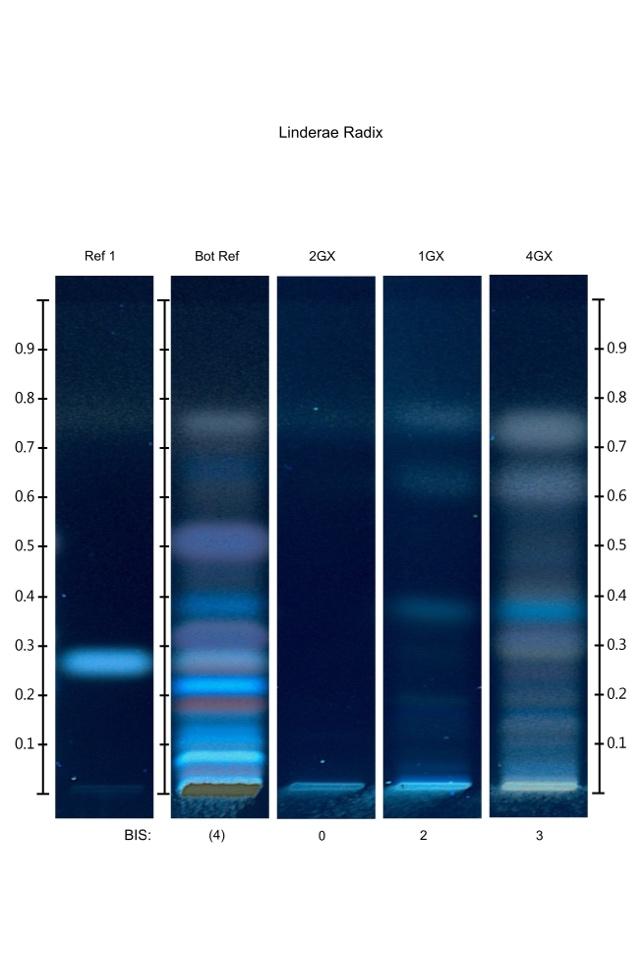
Supplementary Figures S2.D. HPTLC Chromatogram of Linderae Radix with Band Intensity Scores (BISs)

Supplementary Figures S2.E. HPTLC Chromatogram of Corydalis Rhizoma with Band Intensity Scores (BISs)


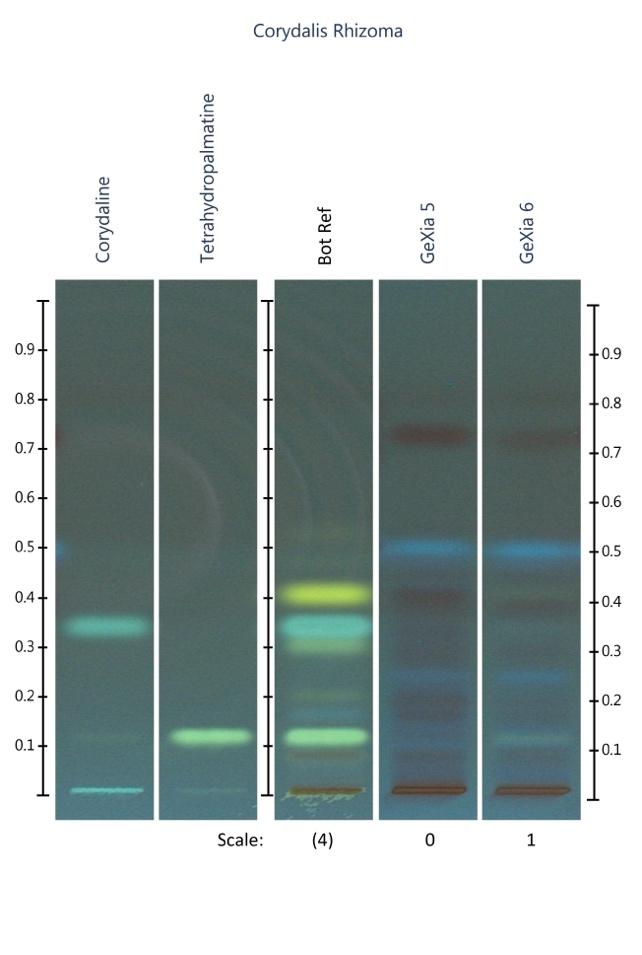


Supplementary Figures S2.F. HPTLC Chromatogram of Chuanxiong Rhizoma with Band Intensity Scores (BISs)


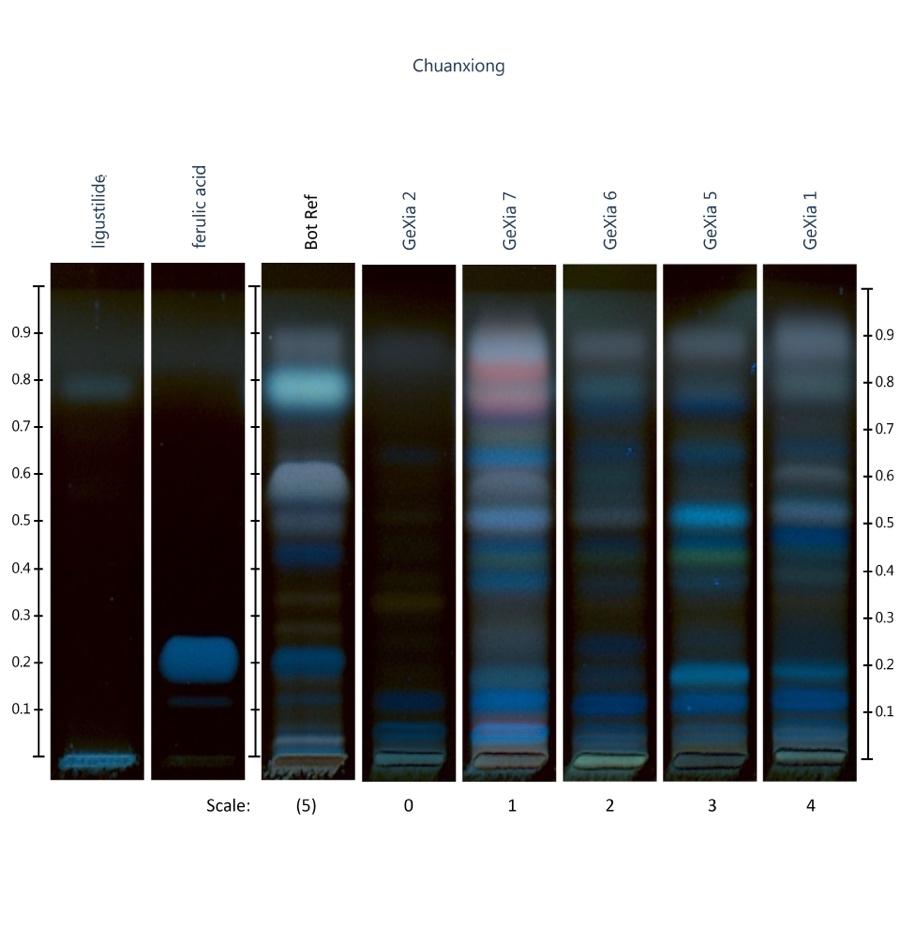


Supplementary Figures S2.G. HPTLC Chromatogram of Glycyrrhizae Radix with Band Intensity Scores (BISs)


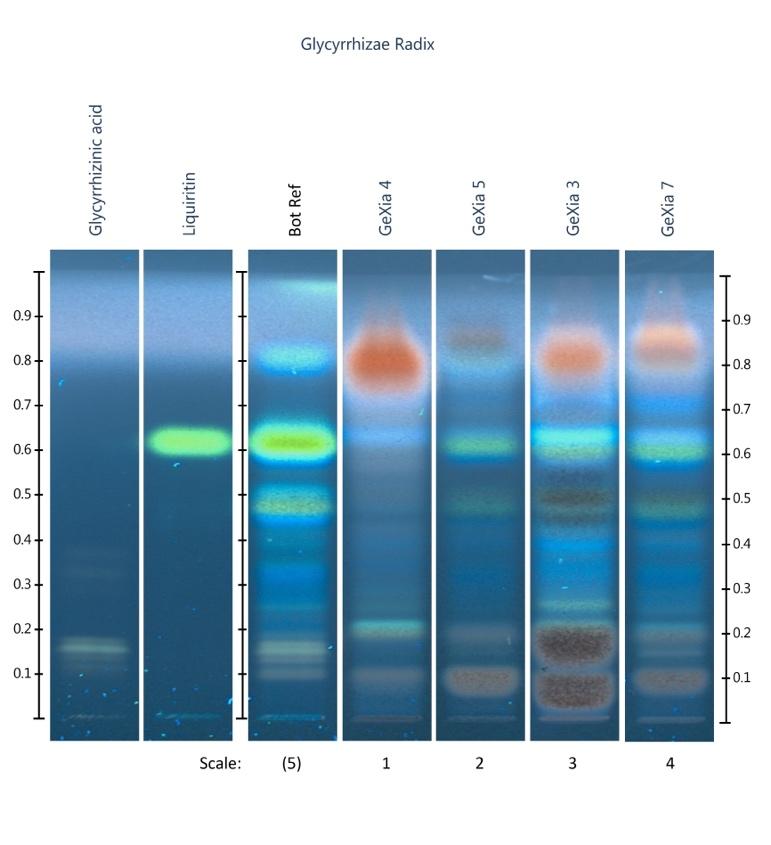


Supplementary Figures S2.H. HPTLC Chromatogram of Cyperi Rhizoma with Band Intensity Scores (BISs)


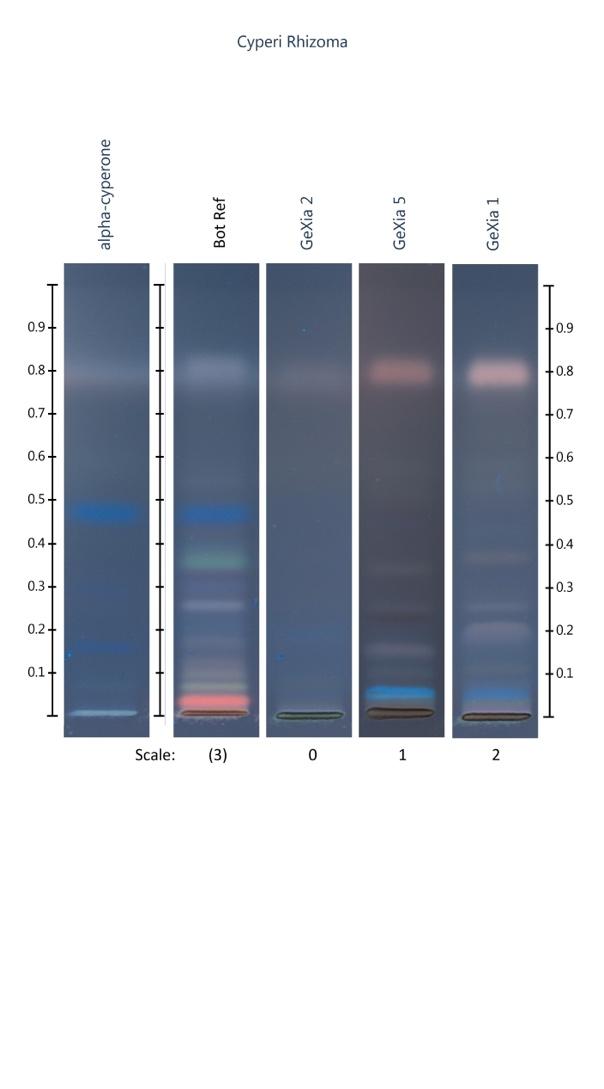


Supplementary Figures S2.I. HPTLC Chromatogram of Auranti Fructus with Band Intensity Scores (BISs)
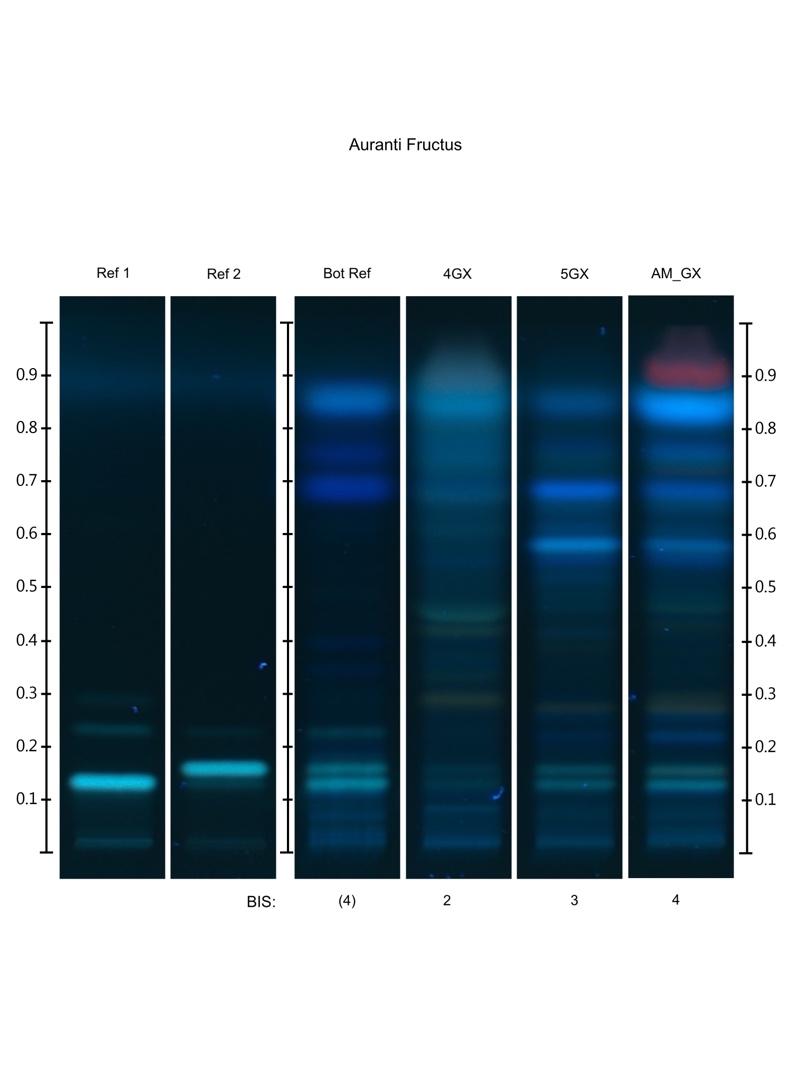


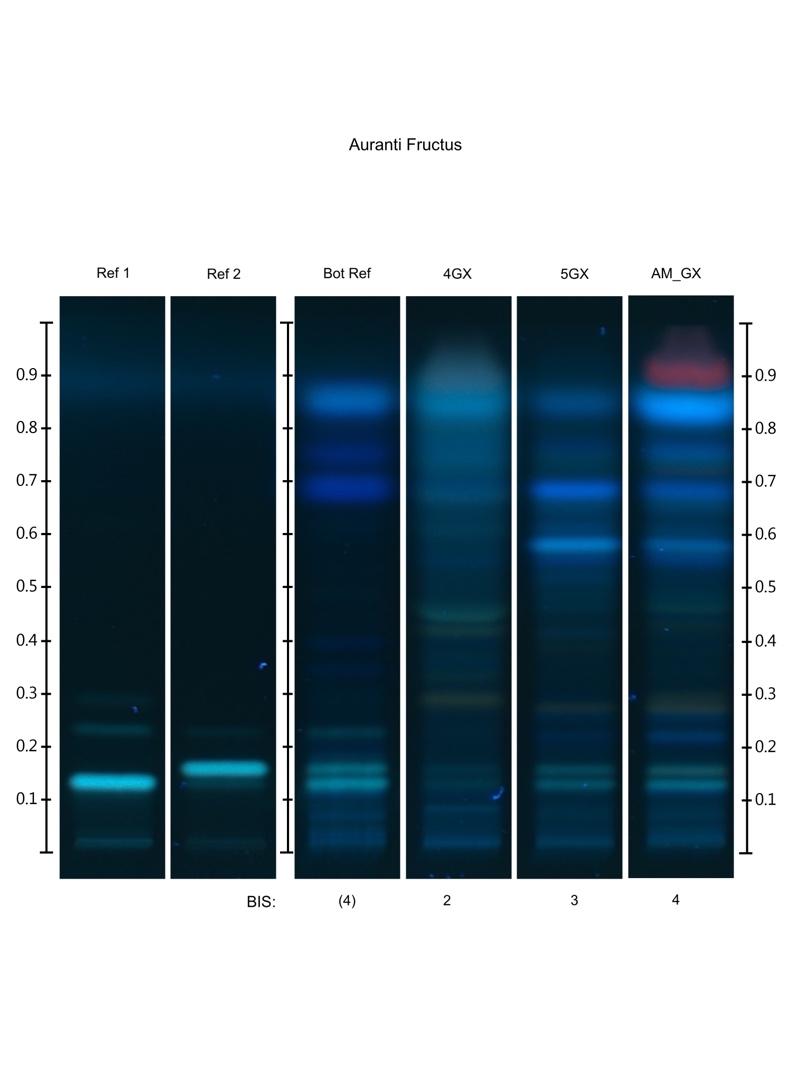

Supplement: Supplementary file 1 [file DataSheet1.zip › Figure 2.docx]
