# Supplementary material for: Complementary authentication of Chinese herbal products to treat endometriosis using DNA metabarcoding and HPTLC shows a high level of variability
Source: Front Pharmacol. 2023 Dec 5;14:1305410. doi: 10.3389/fphar.2023.1305410 (PMC10728824; doi:10.3389/fphar.2023.1305410)

Supplementary Figures S3.A. Metabarcoding results for ITS1, formula FL


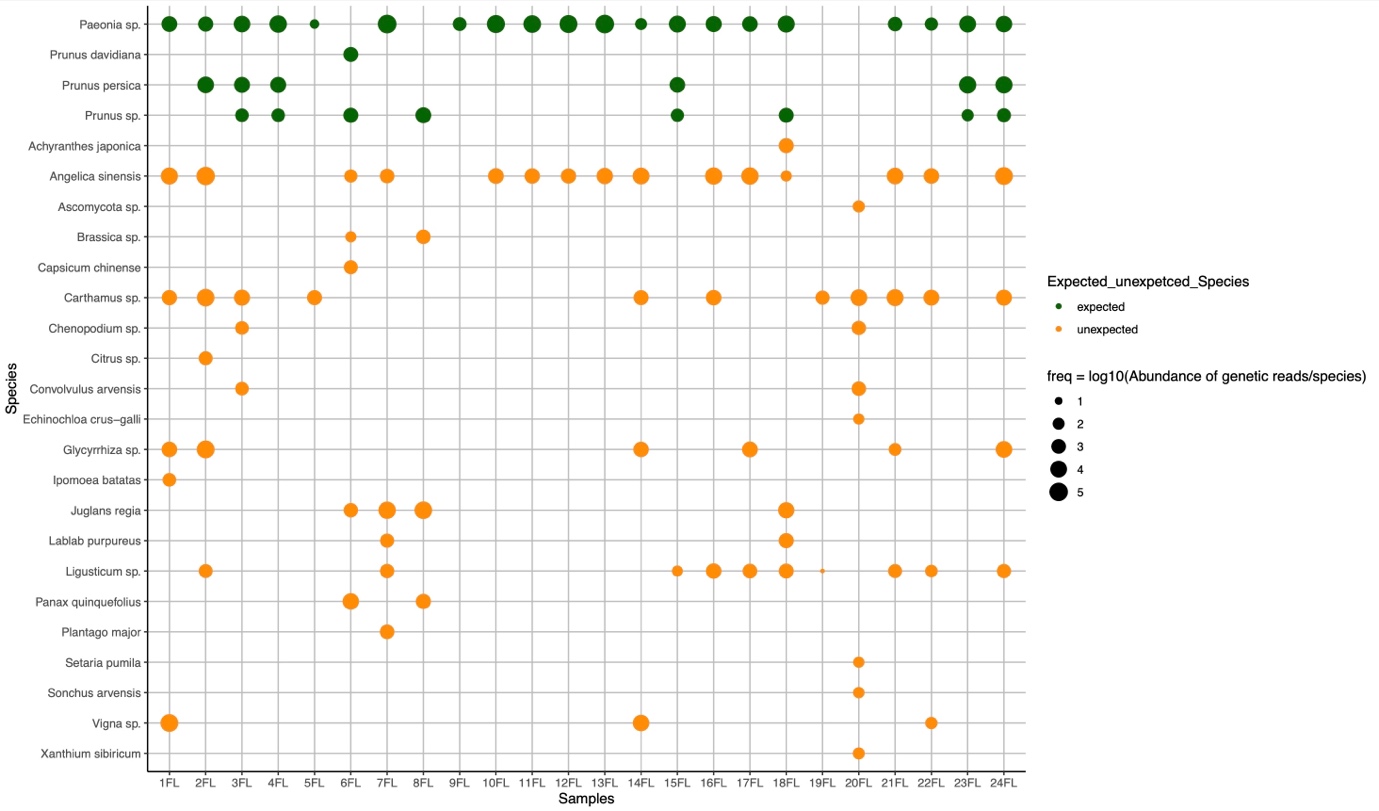


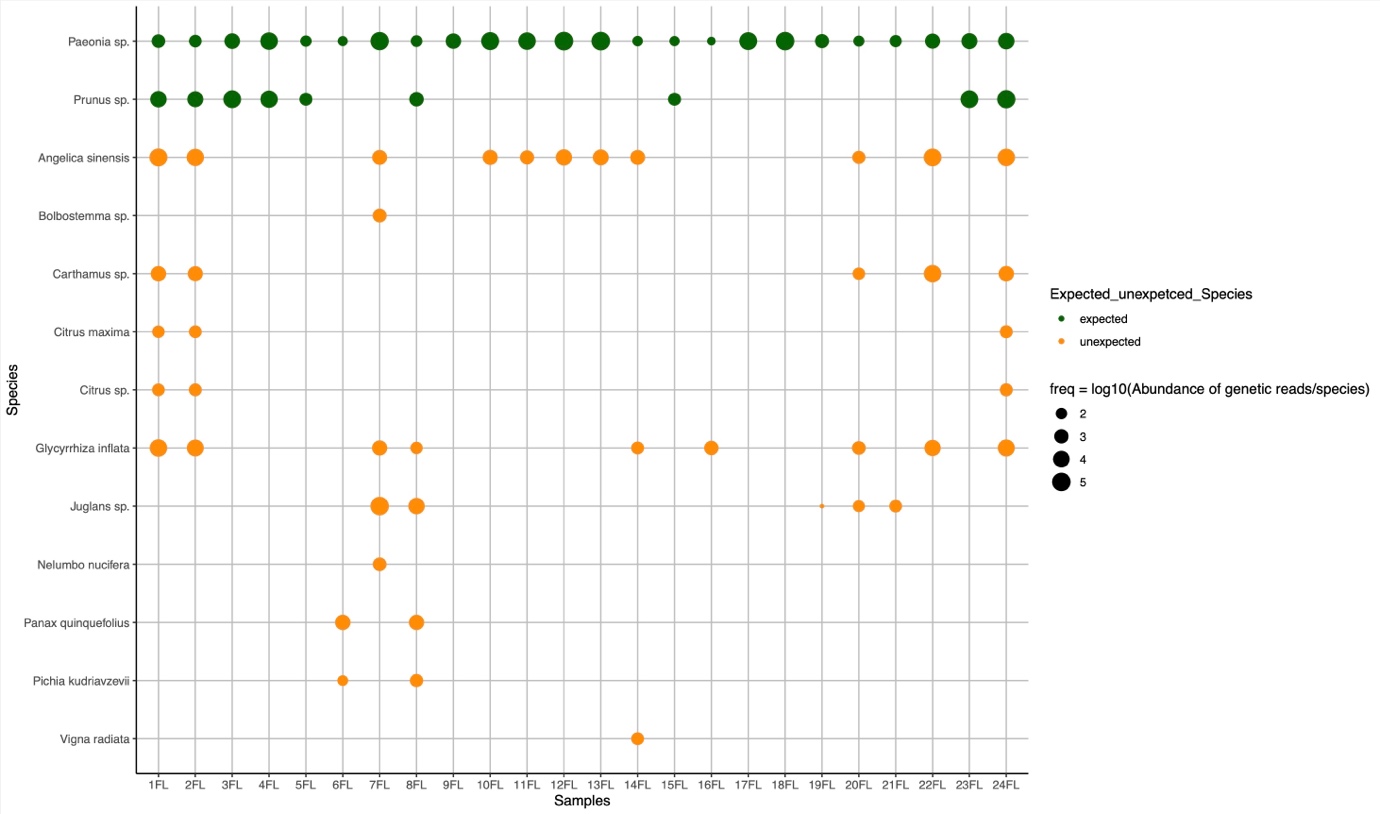


Supplementary Figure S3.B. Metabarcoding results for ITS2, formula FL

Supplementary Figure S3.C. Metabarcoding results for ITS1, formula GX
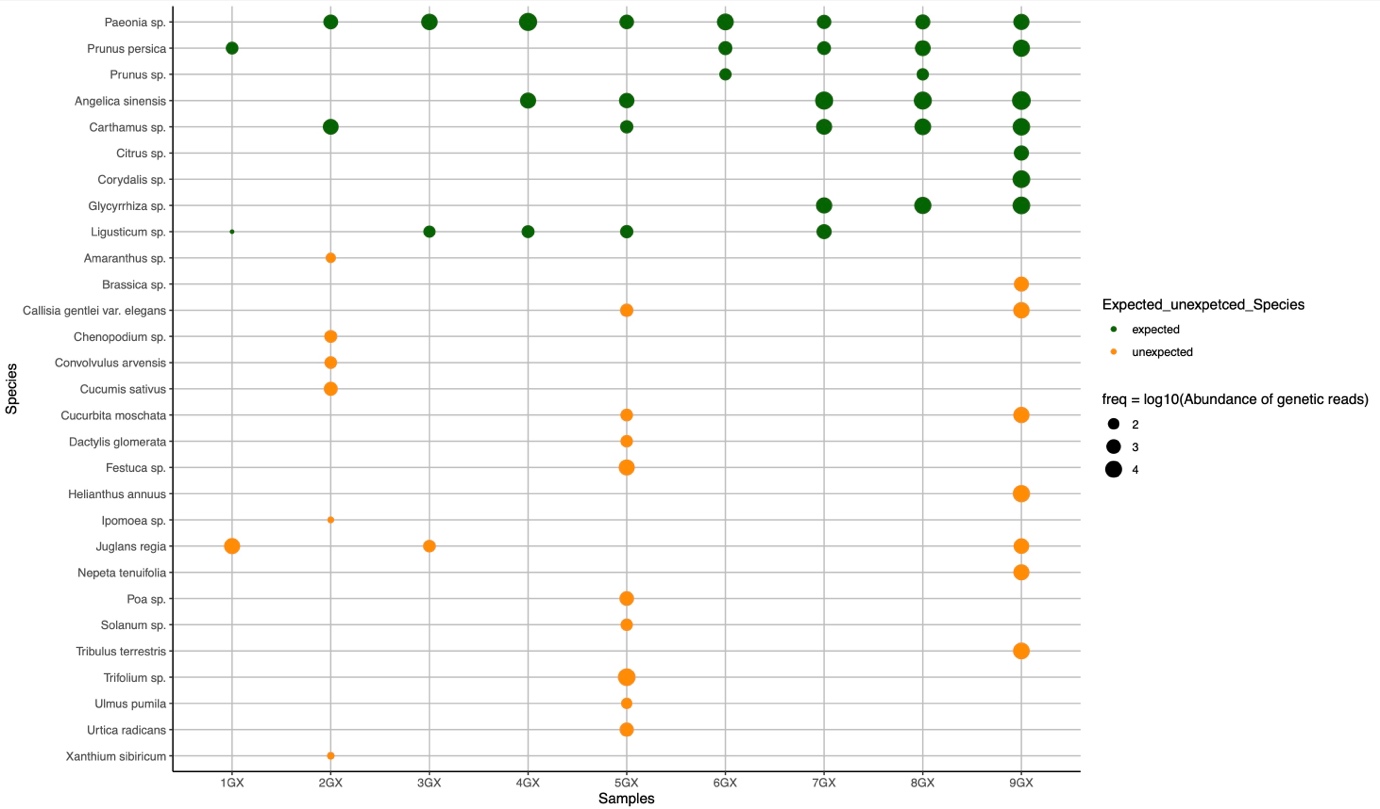


Supplementary Figure S3.D. Metabarcoding results for ITS2, formula GX
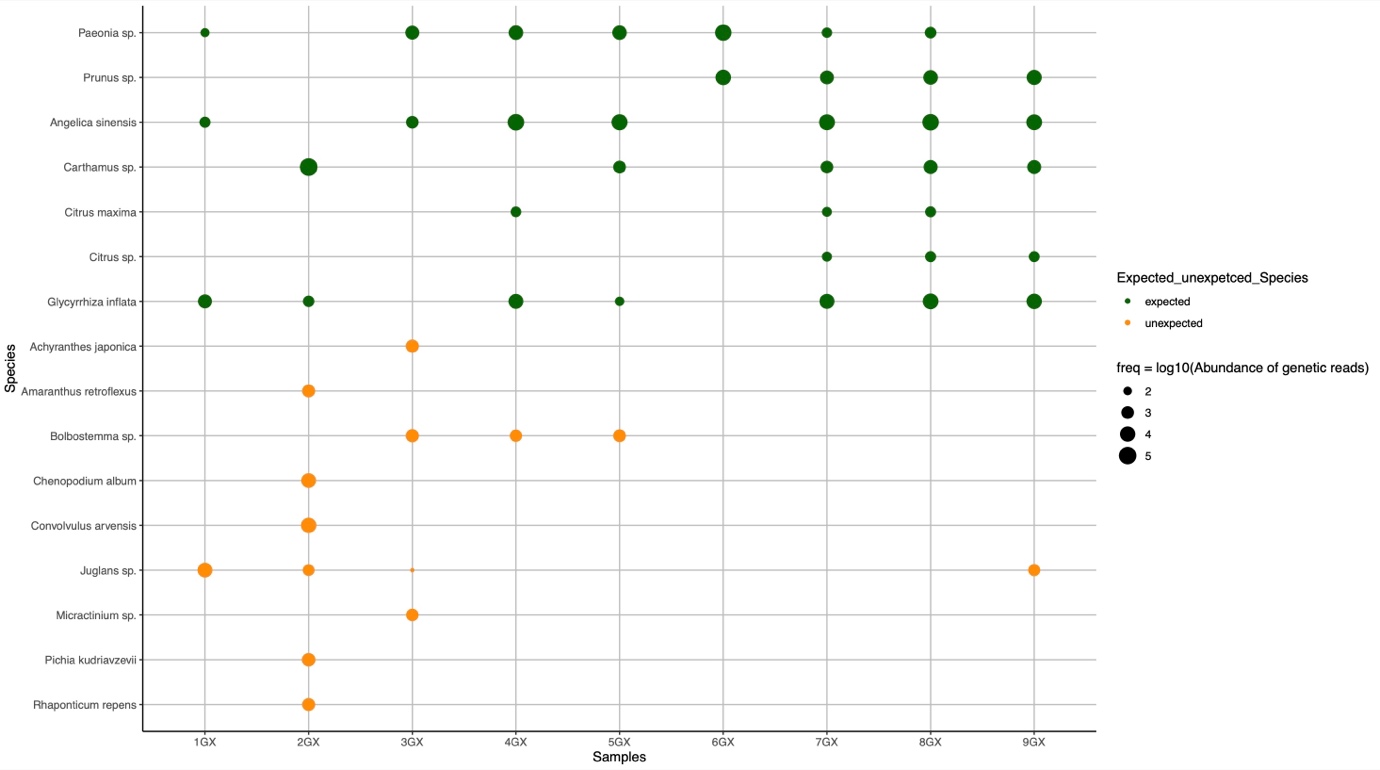

Supplement: Supplementary file 1 [file DataSheet1.zip › Figure 3.docx]
