# Supplementary material for: Complementary authentication of Chinese herbal products to treat endometriosis using DNA metabarcoding and HPTLC shows a high level of variability
Source: Front Pharmacol. 2023 Dec 5;14:1305410. doi: 10.3389/fphar.2023.1305410 (PMC10728824; doi:10.3389/fphar.2023.1305410)

**Only the labeled columns show the fingerprints of samples which are part of this study**

Supplementary Figure S4.A. HPTLC Chromatogram of Cinnamomum Ramulus


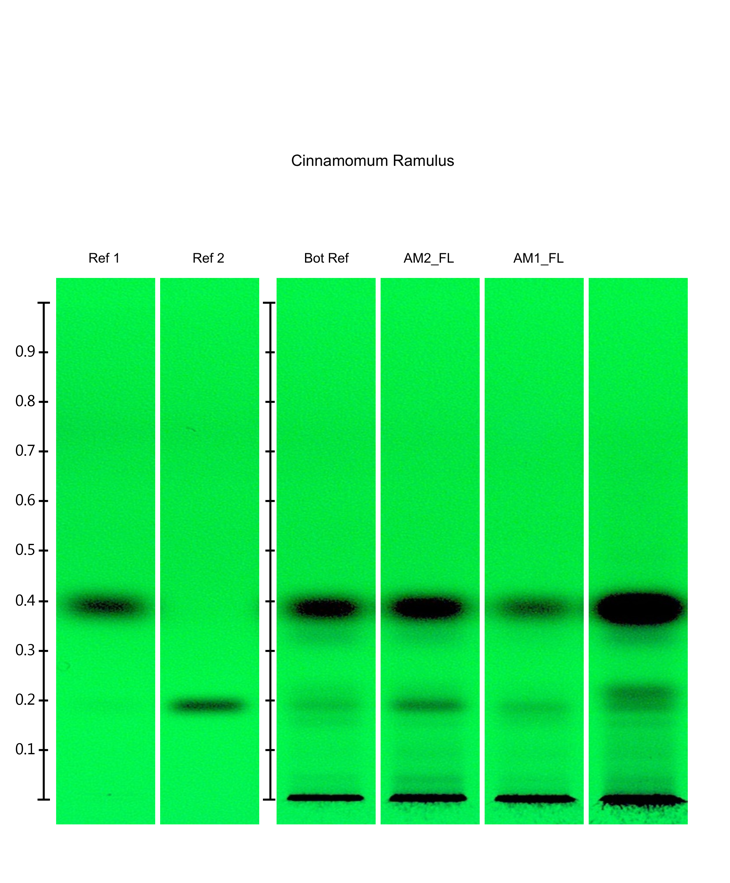


Supplementary Figure S4.B. HPTLC Chromatogram of Moutan Cortex and Paeoniae Radix rubra

Since both ingredients are part of each, formula FL and GX, we here label the fingerprints for samples AM_GX, AM1_FL and AM2_FL.


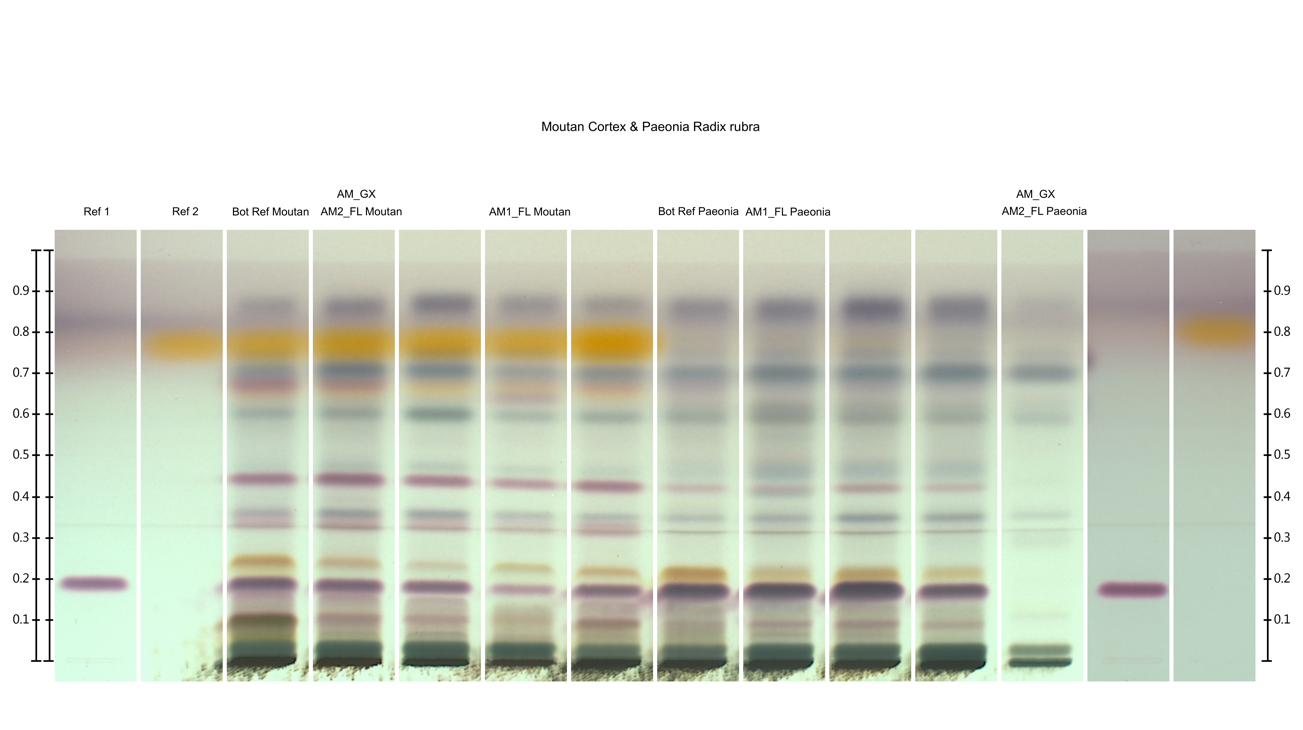


Supplementary Figure S4.C. HPTLC Chromatogram of Chuan Xiong Rhizoma


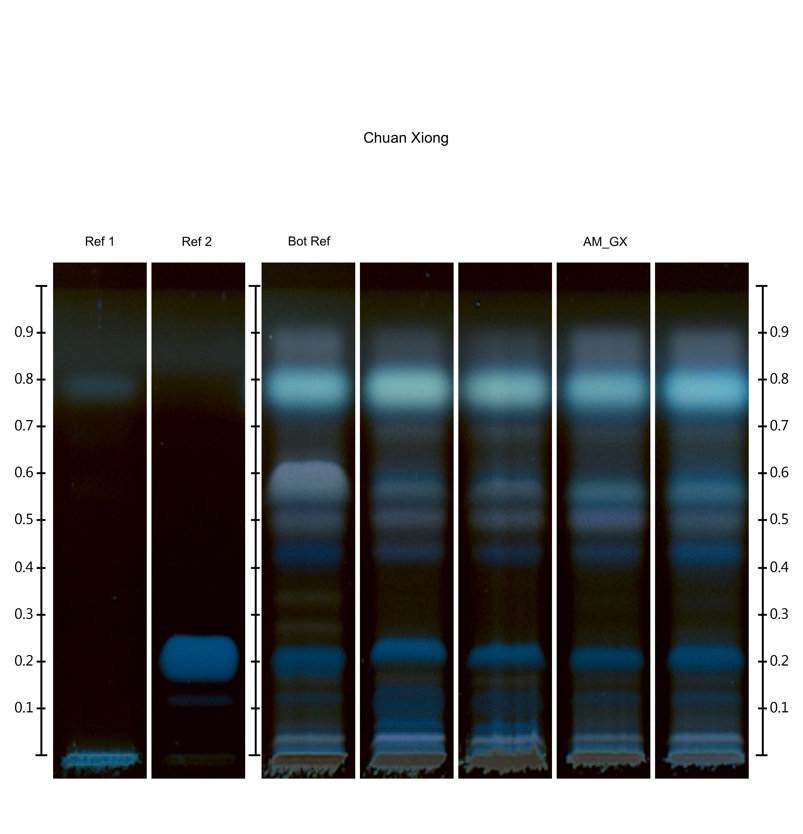


Supplementary Figure S4.D. HPTLC Chromatogram of Carthami Flos


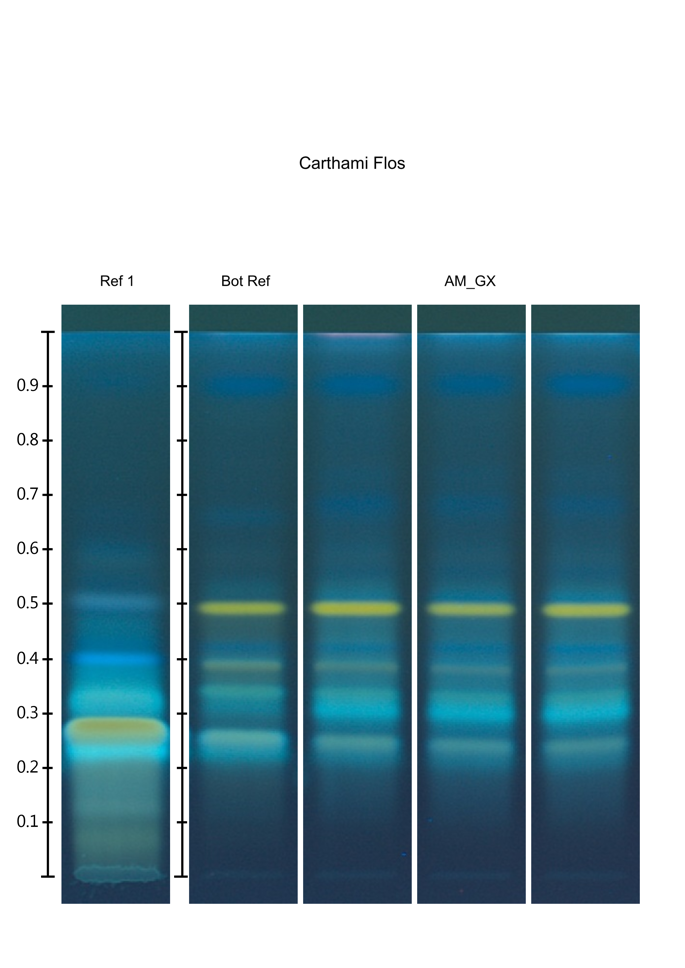


Supplementary Figure S4.E. HPTLC Chromatogram of Poria Cocos


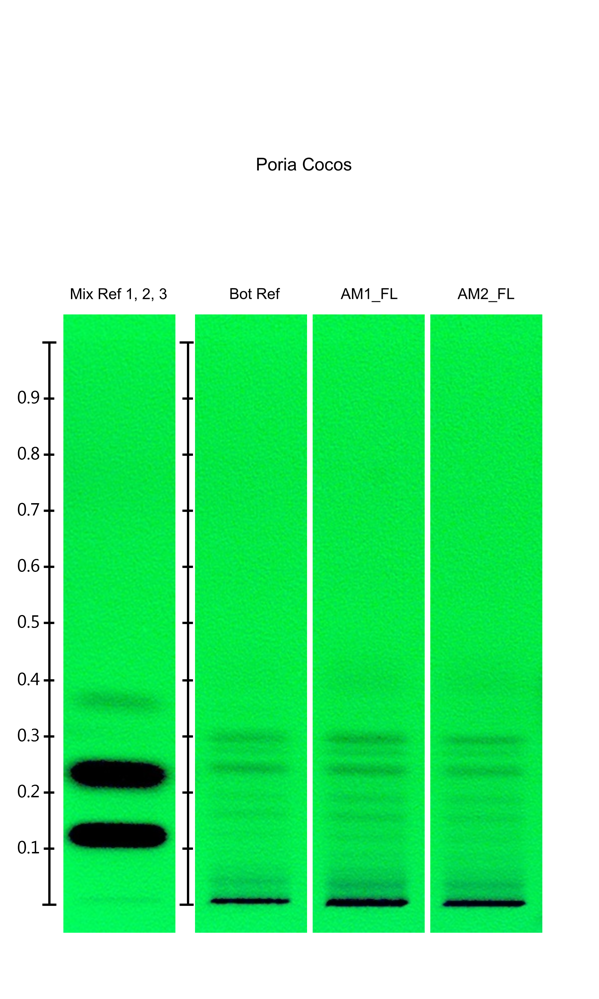


Supplementary Figure S4.F. HPTLC Chromatogram of Persica Semen

Since both ingredients are part of each formula, both AM_GX and AM2_FL represent Persica Semen in the last column.


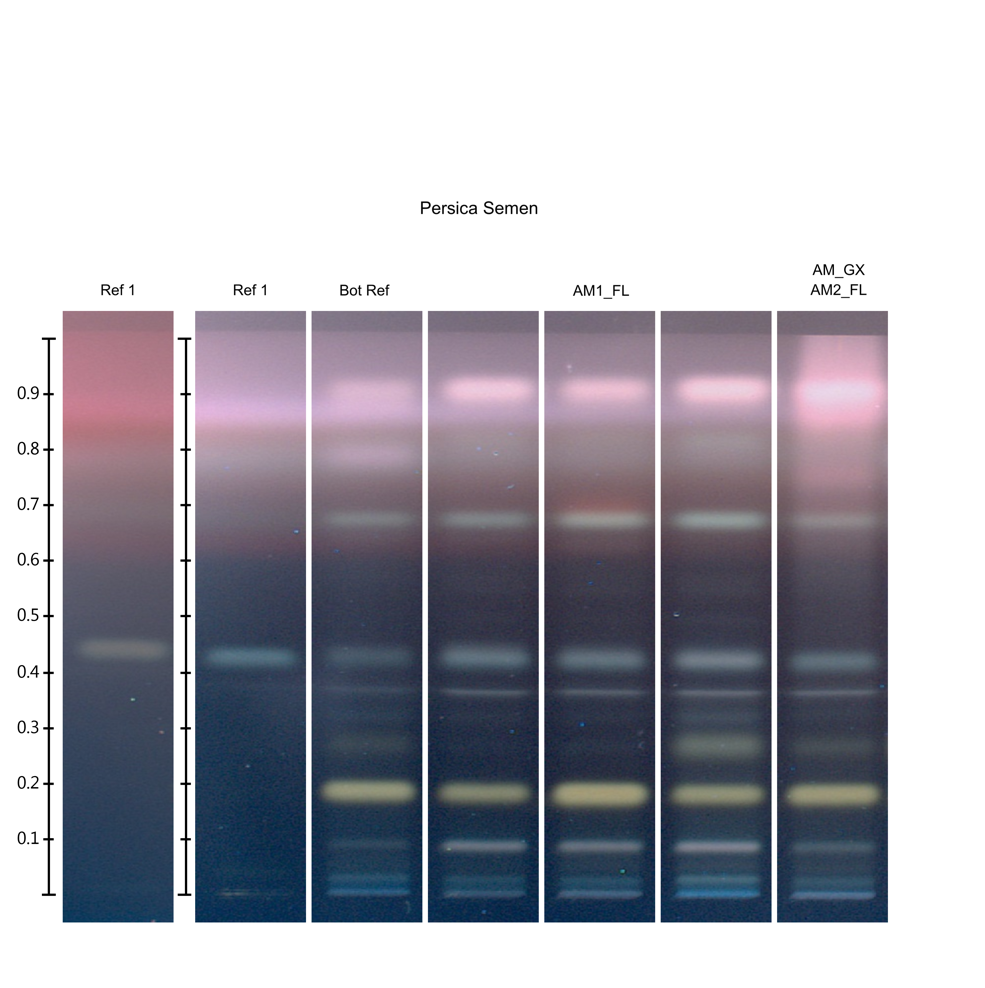


Supplementary Figure S4.G. HPTLC Chromatogram of Angelica Sinensis Lipid Fraction


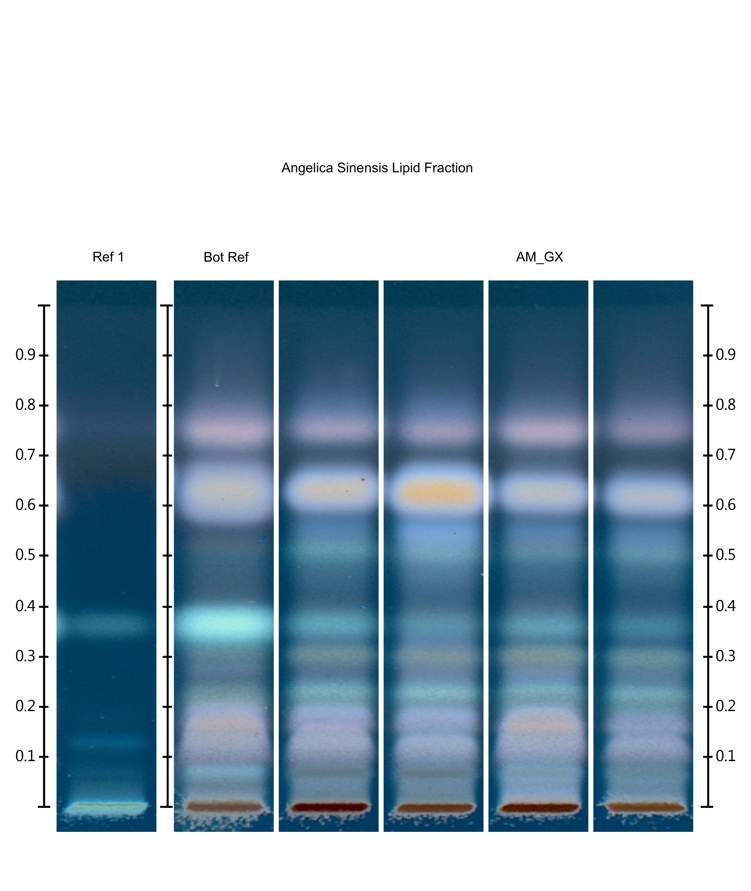


Supplementary Figure S4.H. HPTLC Chromatogram of Angelica Sinensis Water Fraction


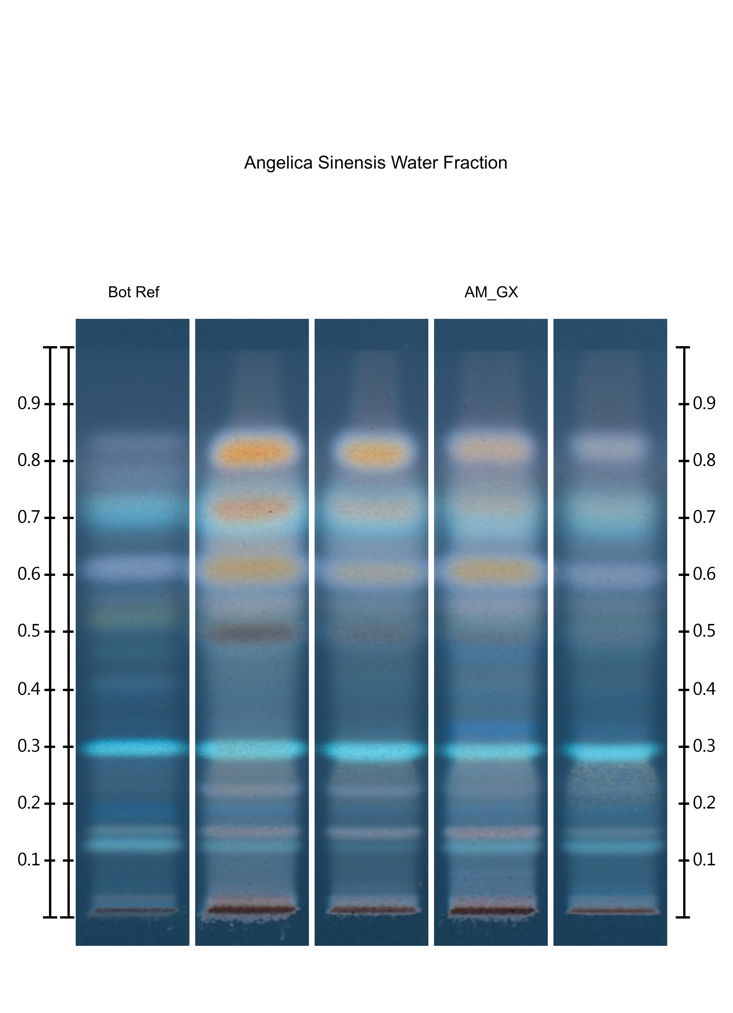


Supplementary Figure S4.I. HPTLC Chromatogram of Auranti Fructus


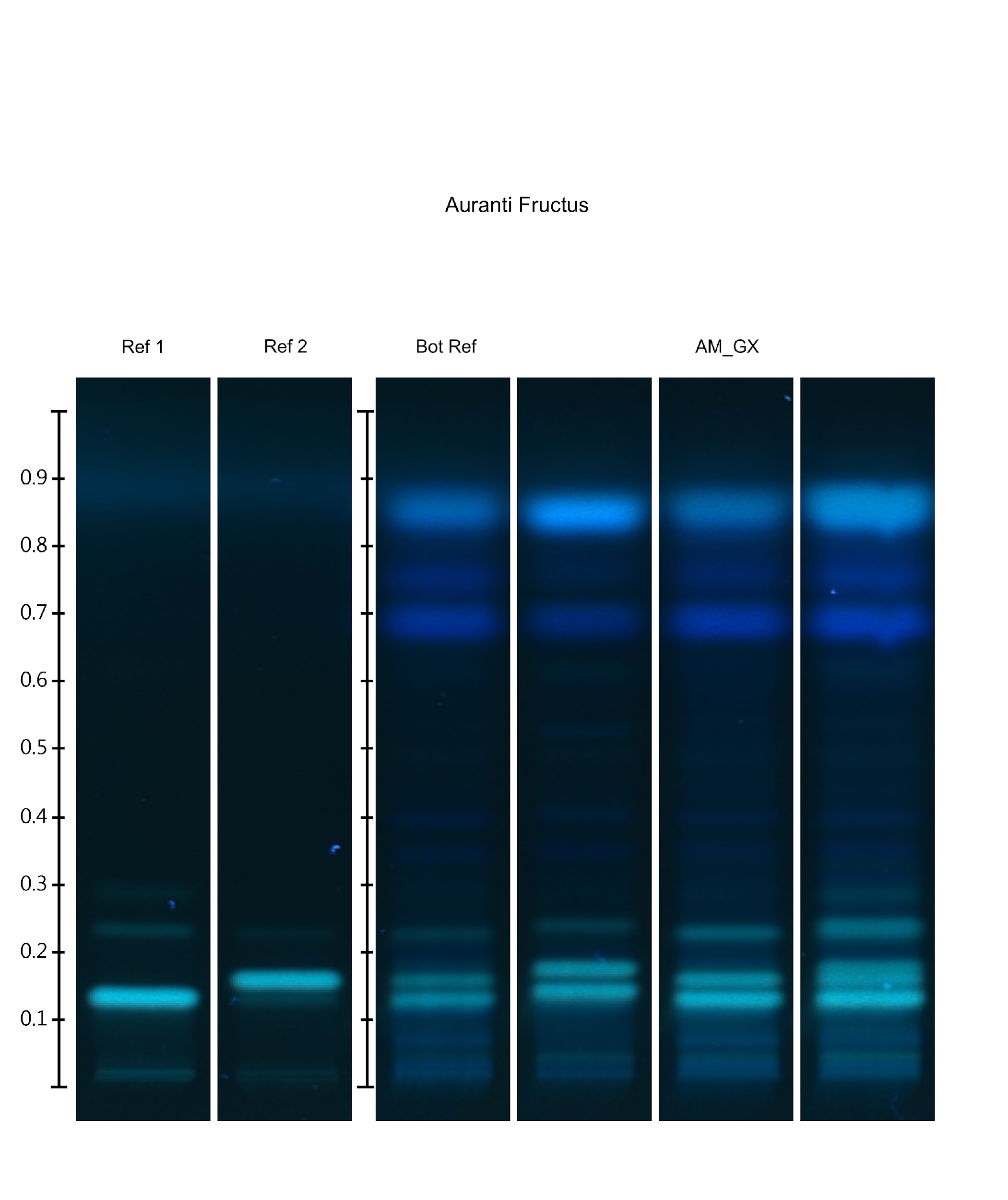


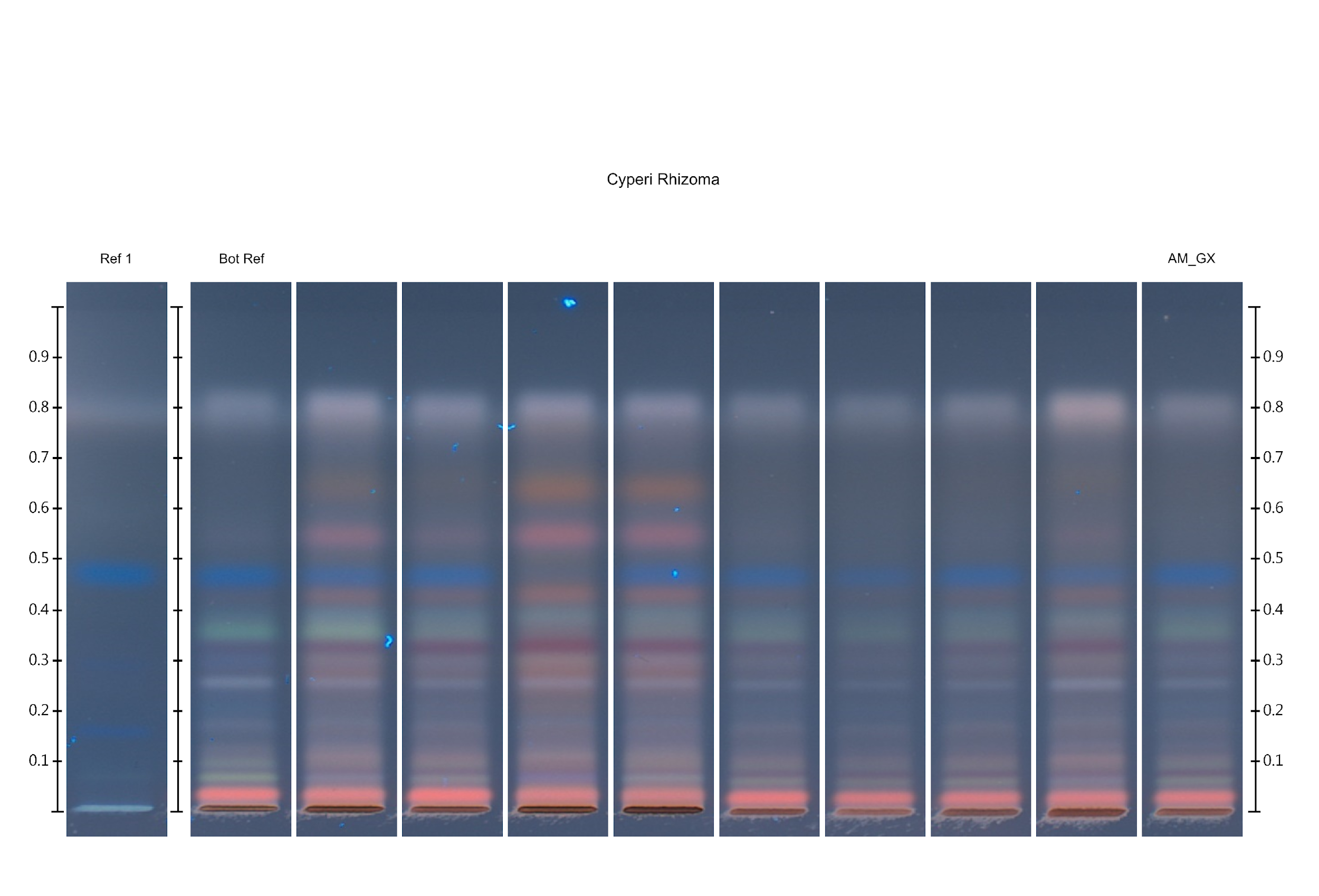
Supplementary Figure S4.J. HPTLC Chromatogram of Cyperi Rhizoma

Supplementary Figure S4.K. HPTLC Chromatogram of Glycyrrhizae Radix


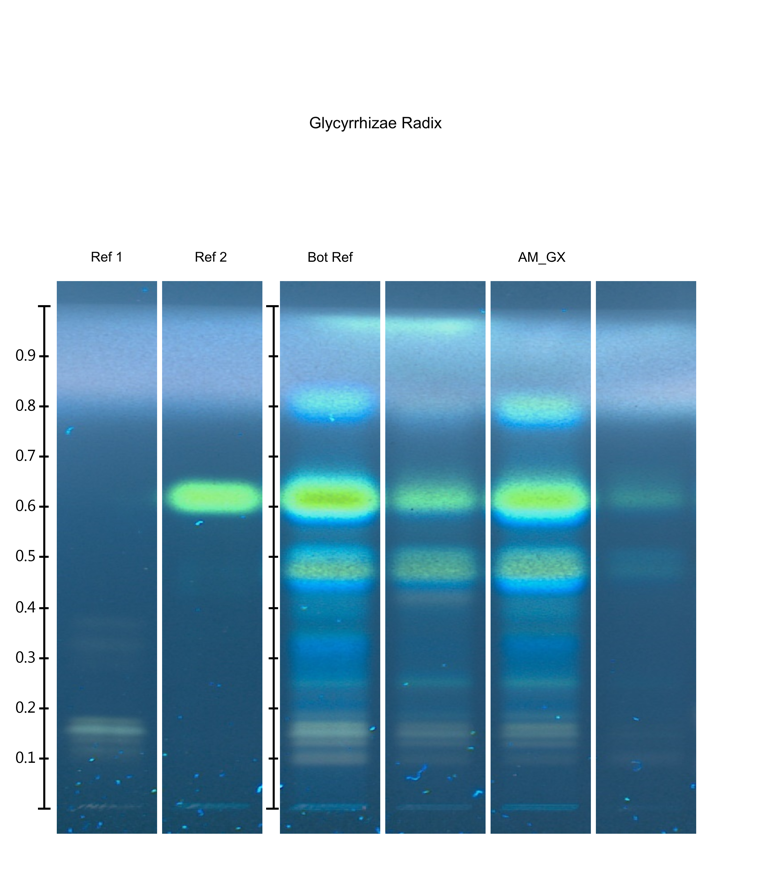


Supplementary Figure S4.L. HPTLC Chromatogram of Linderae Radix


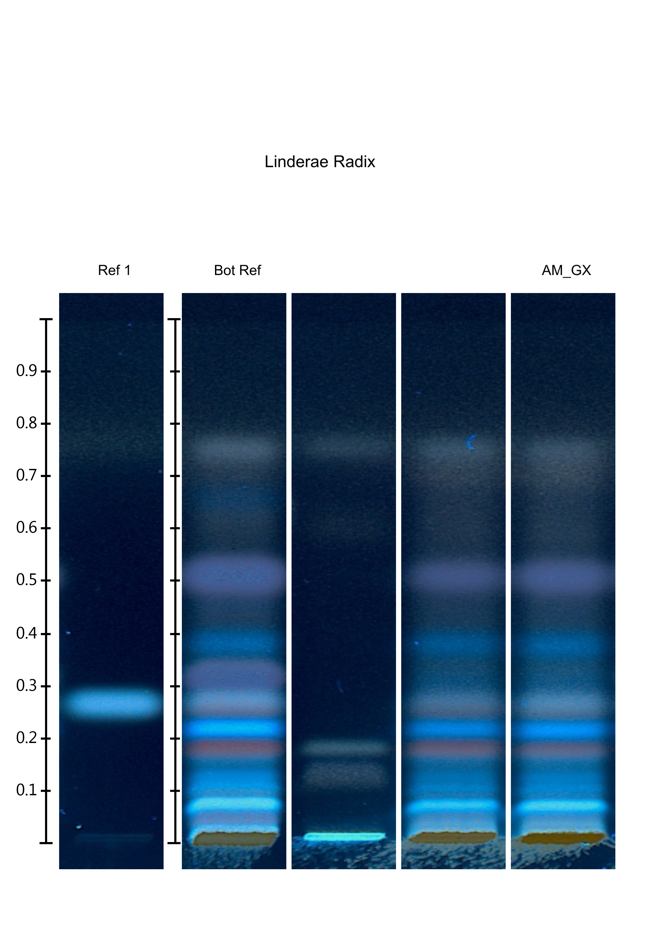


Supplementary Figure S4.M. HPTLC Chromatogram of Corydalis Rhizoma


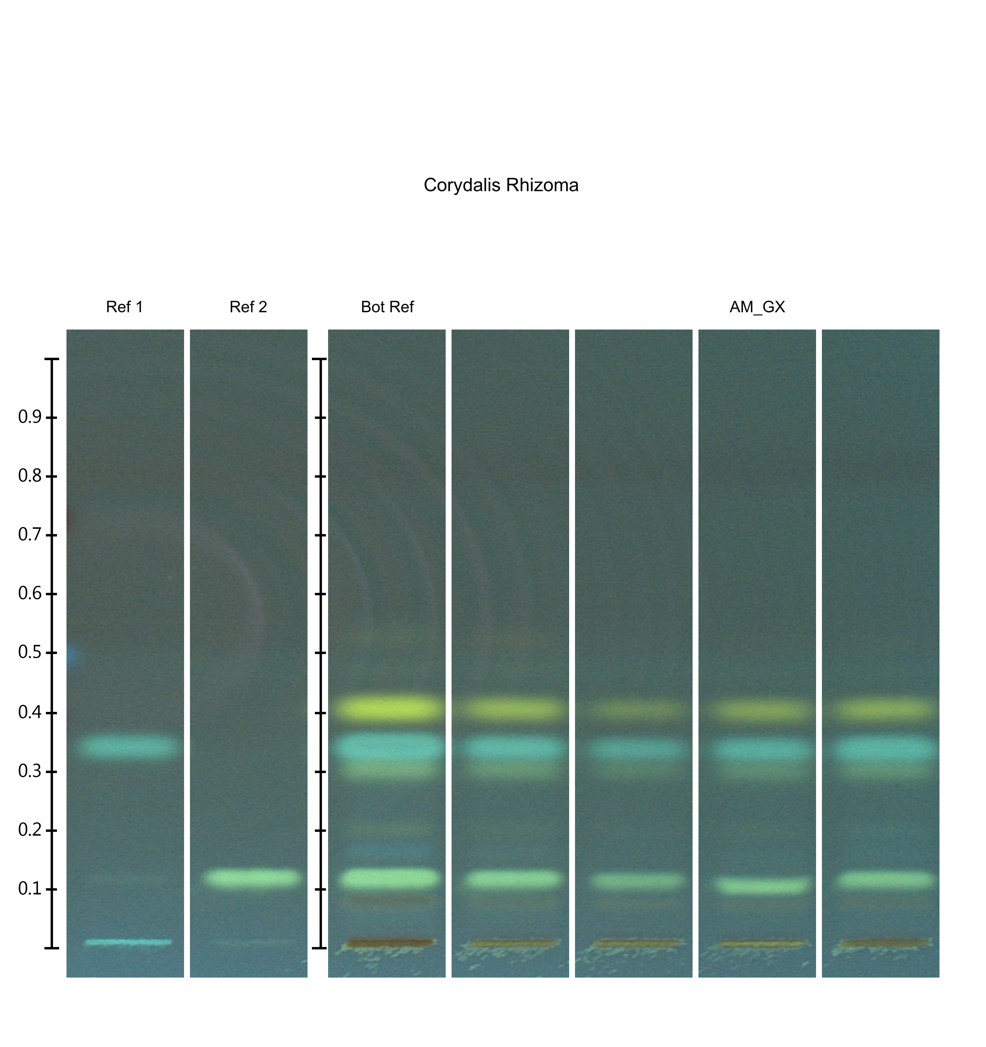

Supplement: Supplementary file 1 [file DataSheet1.zip › Figure 4.docx]
